# Diffusion Nuclear Magnetic Resonance Measurements on Cationic Gold (I) Complexes in Catalytic Conditions: Counterion and Solvent Effects

**DOI:** 10.3390/molecules29133018

**Published:** 2024-06-26

**Authors:** Filippo Campagnolo, Eleonora Aneggi, Walter Baratta, Talha Munir, Daniele Zuccaccia

**Affiliations:** Dipartimento di Scienze Agroalimentari, Ambientali e Animali, Sezione di Chimica, Università di Udine, Via Cotonificio 108, I-33100 Udine, Italy; campagnolo.filippo@spes.uniud.it (F.C.); eleonora.aneggi@uniud.it (E.A.); walter.baratta@uniud.it (W.B.); munir.talha@spes.uniud.it (T.M.)

**Keywords:** gold catalysis, ion pairing, diffusion measurements

## Abstract

The amount of free ions, ion pairs, and higher aggregate of the possible species present in a solution during the gold(I)-catalyzed alkoxylation of unsaturated hydrocarbon, i.e., ISIP (inner sphere ion pair) [(NHC)AuX] and OSIP (outer sphere ion pairs) [(NHC)Au(TME)X] [NHC 1,3-bis(2,6-di-isopropylphenyl)-imidazol-2-ylidene; TME = tetramethylethylene (2,3-bis methyl-butene); X^−^ = Cl^−^, BF_4_^−^, OTf^−^; and OTs^−^ BArF_4_^−^ (ArF = 3,5-(CF_3_)_2_C_6_H_3_)], has been determined. The ^1^H and ^19^F DOSY NMR measurements conducted in catalytic conditions indicate that the dissociation degree (α) of the equilibrium ion pair/free ions {[(NHC)Au(TME)X] 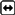 [(NHC)Au(TME)]^+^ + X^−^} depends on the nature of the counterion (X^−^) when chloroform is the catalytic solvent: while the compounds containing OTs^−^ and OTf^−^ as the counterion gave a low α (which means a high number of ion pairs) of 0.13 and 0.24, respectively, the compounds containing BF_4_^−^ and BArF_4_^−^ showed higher α values of 0.36 and 0.32, respectively. These results experimentally confirm previous deductions based on catalytic and theoretical data: the lower the α value, the greater the catalytic activity because the anion that can activate methanol during a nucleophilic attack, although the lower propensity to activate methanol of BF_4_^−^ and BArF_4_^−^, as suggested by the DFT calculations, cannot be completely overlooked. As for the effect of the solvent, α increases as the dielectric constant increases, as expected, and in particular, green solvents with high dielectric constants show a very high α (0.90, 0.84, 0.80, and 0.70 for propylene carbonate, γ-valerolactone, acetone, and methanol, respectively), thus confirming that the moderately high activity of NHC-Au-OTf in these solvents is due to the specific effect of polar functionalities (O-H, C=O, O-R) in activating methanol. Finally, the DOSY measurements conducted in p-Cymene show the formation of quadrupole species: under these conditions, the anion can better exercise its ‘template’ and ‘activating’ roles, giving the highest TOF.

## 1. Introduction

Gold(I) catalysis is a vitally important area of research with many reported examples [1,2,3,4,5,6,7,8,9,10,11], and the mechanistic proposals for reactions involving gold(I) catalysts are well established when concerning the roles of ligands [12,13,14], counterions [15,16,17,18,19,20,21,22,23,24,25], substrates [26,27,28,29,30,31], nucleophiles, solvents, and additives [32].

Over the last few years, some of us has been engaged in the rationalization, from an experimental and theoretical point of view, of the important features of gold(I) [33,34,35,36,37,38,39] and gold(III) [40,41] catalysis. 

We started with the preliminary determination of the ion-pairing structure of [(L)Au(UHS)]^+^X^−^ (L = carbene and phosphane, UHS = unsaturated hydrocarbon, and X^−^ = weakly coordinating counterion) systems, which are the most important intermediates formed during gold-catalyzed nucleophilic additions to an unsaturated substrate. The anion, in order to influence the kinetics of the reaction, must be in the correct position, at least at the RDS of the reaction [42]. From 2009 onwards [43], several interionic characterizations [44,45] of the [(L)Au(UHS)]^+^X^−^ species have been made by some of us researchers, taking advantage of nuclear Overhauser effect (NOE) NMR experiments and DFT calculations of potential energy surfaces (PESs) as well as the Coulomb potential of ions. Recently, also, a cationic gold (III) pre-catalyst ion-pairing structure was determined for the first time using the same approach [46]. These powerful experimental and theoretical methods were used by us to understand the relative anion–cation orientation determined by the nature of the ancillary ligand (L), substrate (S), and counterion (X^−^). This fine-tuning of the interionic structure has paved the way for larger control over the properties and activity of these catalysts [47].

The hydration and alkoxylation of alkynes are key processes for the industrial production of carbonyl derivatives, and the pivotal role of ion pairing in the mechanism of the hydration and alkoxylation of alkynes promoted by the gold(I) catalyst L-Au-X is deeply analyzed and discussed in the literature [48].

**Figure 1:**
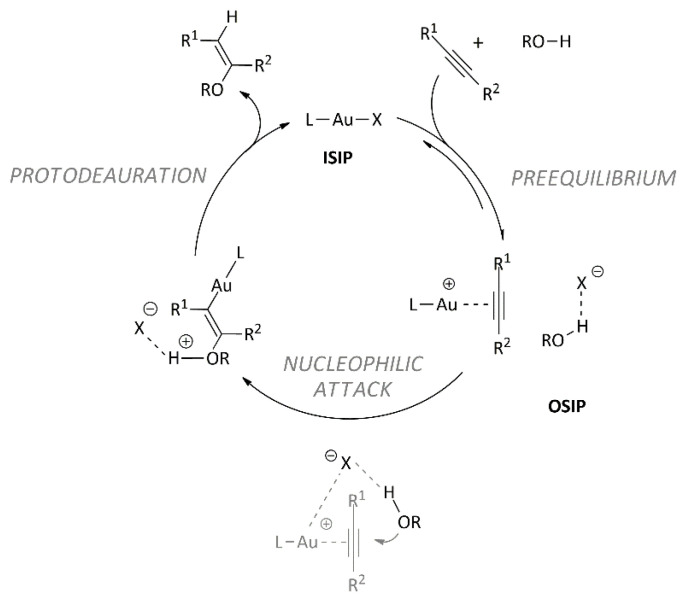
The generally accepted reaction mechanism for gold(I)-complex-catalyzed alkoxylation of alkynes.

Kinetic experiments, together with multinuclear and multidimensional NMR measurements and DFT calculations, allowed us to study, understand, and rationalize the importance of both counterions [49] (in terms of the gold–counterion coordination ability and basicity/proton affinity) and ligands [50,51] (in terms of the donation and π-back donation properties versus gold) in the catalytic cycle (pre-equilibrium step, nucleophilic attack, protodeauration), as shown in Figure 1. Moreover, we have pointed out the crucial role of solvent and noncovalent interactions from both an experimental and theoretical point of view [52]. These results allowed us to develop, for the first time, a green strategy for the hydration of alkynes promoted by gold(I) species in both neat conditions [53] and in green solvents [54,55].

In summary, the main previous results [56] concerning the pivotal role of the counterion in the alkoxylation of alkynes promoted by the L-Au(I) catalyst are as follows:

- The rate-determining step resulted in the nucleophilic attack of methanol on the coordinated alkyne (Figure 1), and the intermediate coordination ability, basicity, and hydrogen bond-accepting properties of OTs^−^ and OTf^−^ provide the best compromise for achieving an efficient catalyst (high TOF) [57].
- In the optimized geometry of the transition state, the anions OTs^−^ and OTf^−^ are located near the alkyne, interacting both with the metal center and with the methanol acting as a template, helping the methanol to assume its reactive position and activating the methanol through a hydrogen bond (enhancing the nucleophilicity of the alcohol). If the basicity of the anion is too low (BF_4_^−^ and BARF^−^), the template effect is lost, and then the hydrogen bonding with the methanol does not take place [58].
- The polarity of the solvent is crucial in determining the catalytic activity of L-Au(I) complexes because it is related to the amount of ion pairs in the solution. Moreover, peculiar functional groups present in the solvent could promote the nucleophilic attack [54].

These important considerations and deductions in gold catalysis are still missing one fundamental finding: the experimental determination of the number of free ions, ion pairs, and higher aggregates in catalytic conditions as a function of both the counterion and solvent.

In this context, pulsed-field-gradient spin-echo (PGSE) NMR and its implementation of DOSY (diffusion-ordered NMR spectroscopy) are the most effective approaches for the analysis of organometallic compounds in a solution [59,60,61]. PGSE and DOSY provide an accurate estimate of the translational diffusion coefficients (D_t_) of the corresponding organometallic compounds. These coefficients can be interpreted using several approaches, which provide information on the molecular sizes and are used to estimate the hydrodynamic radii of organometallic compounds. These approaches have been highly successful in the characterization of neutral gold molecules [62,63] and salts [64,65].

Even if a number the amount of free ions, ion pairs, and higher aggregates present in catalytic conditions, they are very important parameters for gold(I) catalysis, and their determination can be essential to better understand the mechanism of a catalyst; to the best of our knowledge, there are no examples in the literature [66] of PGSE (or DOSY) measurements conducted in catalytic conditions. 

In this paper, the amount of free ions, ion pairs, and higher aggregates in a solution of (NHC)AuX and [(NHC)Au(TME)X] [NHC 1,3-bis(2,6-di-isopropylphenyl)-imidazol-2-ylidene; TME = tetramethylethylene (2,3-bis methyl-butene); X^−^ = Cl^−^, BF_4_^−^, OTf^−^; OTs^−^ BARF^−^ (BARF = B(3,5-(CF_3_)_2_C_6_H_3_)_4_] have been determined by means of ^1^H and ^19^F DOSY NMR, conducted in the catalytic alkoxylation of unsaturated hydrocarbon conditions. These measurements indicate that the dissociation degree (α) of the equilibrium ion pair/free ions {[(NHC)Au(TME)X] 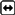 [(NHC)Au(TME)]^+^ + X^−^} and the amount of higher quadrupolar aggregates {2[(NHC)Au(TME)X] 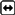 [(NHC)Au(TME)X]_2_} depends on both the nature of the counterion (X^−^) and solvent. These values relate to the performance of the (NHC)Au catalyst as a function of X− and the solvent and provide further insight into the effects of ion pairs in gold catalysis.

## 2. Results and Discussion

**Figure 2:**
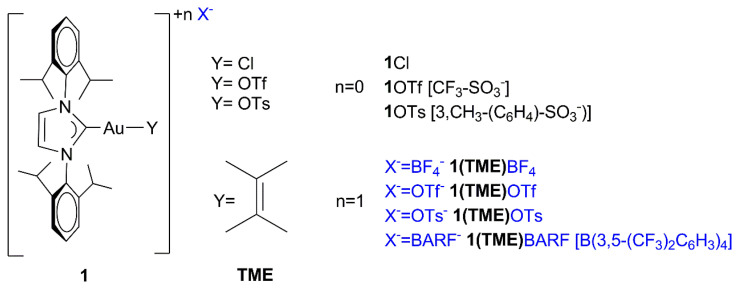
Gold complexes used in this work.

**1**OTf and **1**OTs (Figure 2) have been synthesized following literature-available synthetic protocols [49] by chloride abstraction by adding AgOTf and AgOTs, respectively, to a solution of **1**Cl in methylene chloride, affording the desired compounds with excellent yields and purity (see Section 3 for details). 

On the other hand, **1(TME)**BF_4_ and **1(TME)**BARF have been generated in situ in an NMR tube by chloride abstraction. This was achieved by adding AgBF_4_ and AgBARF, respectively, to (i) a solution of **1**Cl in CDCl_3_ in the presence of five equivalents of TME [TME = tetramethylethylene (2,3-bis methyl-butene)] and (ii) in pseudo-catalytic conditions (solvent, Methanol-d^4^, and TME), affording the desired compounds and AgCl (see Section 3 and Supporting Information for details).

The NMR spectra of complexes **1**OTf and **1**OTs are consistent with those reported in the literature [57]. The NMR spectra of complexes **1(TME)**BF_4_ and **1(TME)**BARF clearly show the characteristic signals of the coordinated alkene, as reported for the compound **1(TME)**SbF_6_ [67].

^1^H and ^19^F DOSY NMR experiments were performed for gold complexes and TME in chloroform-d (Table S2) and in pseudo-catalytic conditions (Table 1 and Table 2). In order to avoid the reaction of 3-hexyne with methanol during the ^1^H and ^19^F DOSY experiments, 3-hexyne was replaced with unreactive TME towards alkoxylation at room temperature. However, this approximation remains valid because the coordination properties towards gold and the dielectric constant of the TME are comparable to those of 3-hexyne.

The experimental observable of DOSY NMR spectroscopy is the self-diffusion coefficients (D_t_, see Section 3) of the species (both cationic, D^+^, or anionic, D^−^), from which the corresponding hydrodynamic radius can be derived by the Stokes–Einstein Equation (1)

(1) $$D_{t}=\frac{kT}{c(r_{solv},r_{H})\pi \eta r_{H}}$$

where k is the Boltzmann constant, T is the absolute temperature, η is the viscosity, and c is a numerical factor, which usually approximates to 6 for large-size molecules.

A more accurate estimation of the hydrodynamic radius r_H_ can be obtained by Equation (2)

(2) $$c(r_{solv},r_{H})=\frac{6}{1+0.695{\left(\frac{r_{solv}}{r_{H}}\right)}^{2.234}}$$

where the c factor is expressed as a function of the solvent-to-solute ratio of radii on the basis of the model proposed by Wirtz and coworkers [68,69], and where r_solv_ is the radius of the solvent. The D_t_ data were treated accordingly to Equation (2), as described in the literature [70], to derive the hydrodynamic dimensions, taking the solvent or TME as the internal standard. Assuming the shape of the aggregate is spherical, r_H_^±^ can easily be converted to a hydrodynamic volume (V_H_^±^). The average degree of aggregation can be evaluated by dividing V_H_^±^ by the hydrodynamic volume of the ion pair, V_H_^0,IP^, in order to derive the aggregation number (N^±^). 

Estimating V_H_^0,IP^ is not straightforward, and relying on the van der Waals and crystallographic volume of the species is unreliable, particularly when aromatic groups are present [71]. By choosing the experimental conditions properly to avoid any ion-pairing process (for example, performing PGSE measurements in a polar solvent at a low concentration [72]), it is possible to measure V_H_^0,IP^ as the sum of the V_H_^0,−^ (the hydrodynamic volume of the free anion) and V_H_^0,+^ (the hydrodynamic volume of the free cation) [72]. If N^±^ are both equal or smaller than 1, the amount of ion triples or quadruples of higher aggregates can be considered small, and ion pairing might be assumed as the only process active in the solution. In such a case, it is useful to define the dissociation degree (α) by Equation (3) in order to quantify the relative concentration of free ions and ion pairs:

V_H_^−^ = α (V_H_^0,−^) + (1 − α) V_H_^0,IP^ (3)

Before carrying out the measurements under pseudo-catalysis conditions, diffusion measurements on compounds **1**OTf, **1**OTs, **1(TME)**BF_4_, **1(TME)**BARF, and TME (which we will use as an internal standard in the pseudo-catalytic measurements) were performed. The obtained values of D_t_, r_H_, and V_H_ are given in the Supporting Information. The hydrodynamic volumes ranged from 732 Å^3^ to 840 Å^3^ for compounds **1**OTf and **1**OTs, in line with the literature data for compound **1**Cl (752 Å^3^). With regard to compound **1(TME)**BF_4_, the values are 800 Å^3^ and 850 Å^3^ for the anion and the cation, respectively, in line with those obtained for **1(4-Me-styrene)**BF_4_ in CD_2_Cl_2_. In addition, a D_t_ (CDCl_3_)/D_t_ (TME) ratio of 0.9 was obtained, together with a value of V_H_ of 100 Å^3^ for TME (considering CDCl_3_ as the internal standard). At this point, we started the measurements under pseudo-catalytic conditions in CDCl_3_ (400 μL of CDCl3, 142 μL of CD_3_OD, and 105 μL of TME). We began by measuring the catalytic mixture with precursor **1**Cl, which is inactive in catalysis (Table 1). The D_t_ (CDCl_3_)/D_t_ (TME) is now equal to 0.88 but very close to 0.90. This means that we can apply the methodology known in the literature, even if a mixture of solvents is used. We, therefore chose TME as an internal standard and r_solv_ of CDCl_3_ (Equation (2)) for the treatment of diffusion measurements and thus calculated the hydrodynamic radii and volumes of the gold catalyst.

The value obtained for **1**Cl was 937 Å^3^. This V_H_ was taken as the reference volume of the cation **1**^+^ (NHC-Au^+^) [73]. We have calculated the V_H_^0,IP^ of **1(TME)**X while considering the additive volumes (**1**^+^, TME and anion) and the literature V_H_^0,−^ values [74], where the results are 1152 Å^3^ (X^−^ = OTf^−^), 1245 Å^3^(X^−^ = OTs^−^), 1089 Å^3^ (X^−^ = BF_4_^−^), and 1927 Å^3^ (X^−^ = BARF^−^). These data were used to obtain the values of the aggregation numbers and α, as described above. Since V_H_^−^ is always much larger than V_H_^0,−^, V_H_^−^ is a more sensitive probe than V_H_^+^ to quantitatively assess the ion pairing in a solution, whereas values of N^±^ larger than 1 indicate the presence of higher aggregates [74]. The experimental error on V_H_^±^, N^±^, and α is estimated as 10% [70].

Table 1 shows the results of the diffusion NMR measurements conducted under the same pseudo-catalytic conditions for the gold complexes as a function of the counterion. The values of the hydrodynamic radii of the cations r_H_^+^ vary from 6.2 Å (entry 3, Table 1) to 6.9 Å (entry 4, Table 1), corresponding to volumes between 998 Å^3^ and 1346 Å^3^. These hydrodynamic volumes (V_H_^+^) are about equal to those of the ion pair (V_H_^0,IP^) for compounds **1(TME)**OTs (entry 2, Table 1) and **1(TME)**OTf (entry 3, Table 1) while the value for complex **1(TME)**BF_4_ (entry 4, Table 1) is slightly higher and the V_H_^+^ of complex **1(TME)**BARF is lower. As far as the V_H_^−^ is concerned, the values range from 720 Å^3^ to 1596 Å^3^ (r_H_^−^ from 5.6 Å to 7.3 Å, respectively) and are less than the respective V_H_^0,IP^. Analysis of the anion aggregation number (N^−^) shows that the values are close to 1 for complexes **1(TME)**OTs and **1(TME)**OTf, while the values are less than 1 for complexes **1(TME)**BF_4_ and **1(TME)**BARF. It can be stated with confidence that complexes **1(TME)**OTs and **1(TME)**OTf have a greater tendency to form ion pairs than complexes **1(TME)**BF_4_ and **1(TME)**BARF. This is in line with what has already been observed in the literature for organometallic complexes and organic salts. 

Having values of an aggregation number less than one to better quantify the amount of ion pairs, we calculated the dissociation degree (α), Equation (3). The (α) values span from 0.13 for **1(TME)**OTs to 0.24 for **1(TME)**OTf and have almost the same values of 0.32 and 0.36 for **1(TME)**BF_4_ and **1(TME)**BARF, respectively. 

**Figure 3:**
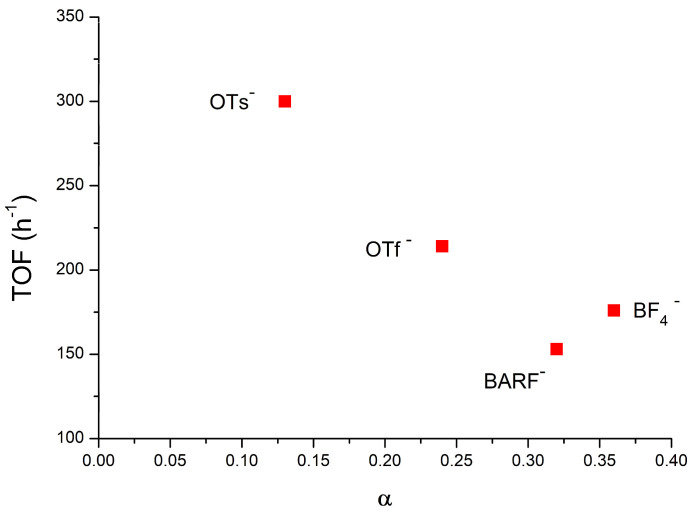
Plot of TOF [TOF (h^−1^) = moles of product/moles of catalyst/time (h)] vs. α for **1(TME)**X in pseudo-catalytic conditions (Table 1). See text for details.

In order to be able to analyze the role of the anion during catalysis in major detail, the trend of the degree of aggregation α against TOF [TOF (h^−1^) = moles of the product/moles of the catalyst/time (h)] is shown in Figure 3 (data are also present in Table 1) [49]. From the analysis of Figure 3, it can be seen that the anion that has the highest TOF value of 300 h^−1^—**1(TME)**OTs—is the one with the lower α, i.e., the highest amount of ion pairs. This is expected because the anion helps during the nucleophilic attack of methanol with a templating effect (helping the methanol to assume its reactive position and activate the methanol through a hydrogen bond). This is only possible with ion pairs and is absent when the catalyst is present in the solution in the form of free ions. 

The combination of the present NMR diffusion results with the previous catalytic and theoretical data allows us to conclude that the different reactivity between **1(TME)**OTs and **1(TME)**OTf is given by both factors: anion OTs^−^ have a higher templating power with respect to OTf^−^, but on the one hand, **1(TME)**OTs is also present in the solution in the form of an ion pair in higher amounts with respect to **1(TME)**OTf. 

The higher α values, together with the low TOF values for anions BF_4_^−^, BARF^−^ 176 h^−1^, and 153 h^−1^, respectively [49], unequivocally demonstrate that the low activity is due to the low percentage of ion pairs in catalytic conditions compared with the anions OTs^−^ and OTf^−^. However, it is important to note that the low templating and activating effect of BF_4_^−^ and BARF^−^, with respect to OTs^−^ (and OTf^−^), cannot be completely neglected: in these conditions, it has been suggested that a second methanol molecule could be involved in the mechanism.

This is the first time that experimental and theoretical catalytic data and NMR diffusion results have been combined for gold catalysis. It allows us to deeply analyze the role of the anion during the rate-determining step of the alkoxylation of alkynes, i.e., the nucleophilic attack (Figure 1) in light of the different amounts of ion pairs and different templating and activating effects of the counterion.

The same methodology (theoretical and experimental kinetic data compared with the NMR diffusion experiments) is the best way to understand the role of the functional groups of solvent and additive in gold catalysis. Gold affinity and hydrogen-bond basicity of the functional groups of additives and solvent are very important topics, being recently the subject of a review by Xu and collaborators [75]; however, further mechanistic studies and novel control experiments are needed for a deeper understanding of the additives and solvent role in gold catalysis. 

Table 2 shows the values of the D_t_ (10^−10^ m^2^s^−1^), r_H_^±^ (Å), V_H_^±^ (Å^3^), aggregation number (N^±^), and α for complexes **1(TME)**OTf in different solvents (p-cymene, chloroform, methanol, acetone, γ-valerolactone, and propylene carbonate). The values of the hydrodynamic radii of the cations r_H_^+^ vary from 6.1 Å (entry 4, Table 2) to 8.4 Å (entry 1, Table 2), corresponding to volumes between 946 Å^3^ and 2509 Å^3^. These hydrodynamic volumes (V_H_^+^) are about equal to those of the ion pair (V_H_^0,IP^) for compounds **1(TME)**OTf in all solvents except p-cymene (entry 1, Table 2). As for V_H_^−^, the values ranged from 217 Å^3^ to 1499 Å^3^ (r_H_^−^ from 3.7 Å to 7.1 Å, respectively) and are less than the respective V_H_^0,IP^, except for the value for p-cymene (entry 1, Table 2)

The values of the aggregation numbers (N^±^) are all less than 1, with the exception of p-cymene (entry 1, Table 2). The values of 2.18 and 1.3 for N^+^ and N^−^, respectively, indicate the formation of aggregates greater than the ion pairs. The values for chloroform, already commented above (entry 3, Table 1), indicate a high presence of ion pairs with respect to free ions, whereas the very low values of N^−^ for the other solvents indicate a very low aggregation of ions, as expected due to their high dielectric constant. 

Having values of an aggregation number of less than one (entries 2–6, Table 2) to better quantify the amount of ion pairs, we calculated the dissociation degree (α), Equation (3). For p-cymene, we can confidently assume that the α value is zero, as the values are greater than 1 and there are no free ions from the ion-pair/free-ions equilibrium.

The (α) values spanned from 0 for p-cymene to 0.90 for propylene carbonate, and almost the same higher values of 0.70, 0.74, and 0.80 for methanol, acetone, and γ-valerolactone, respectively, were obtained.

**Figure 4:**
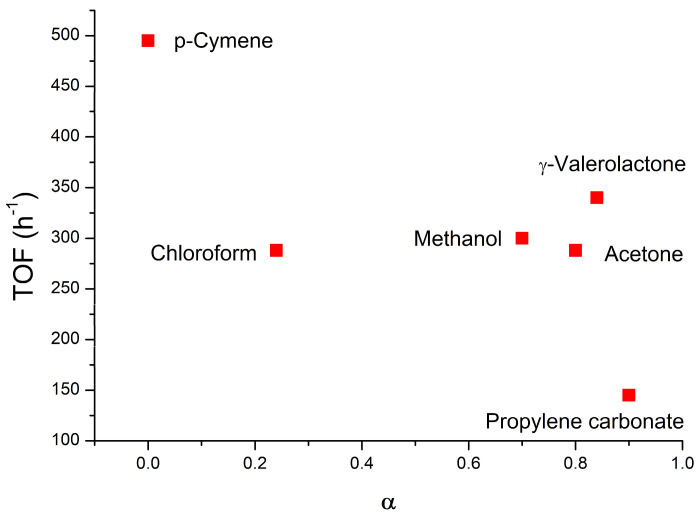
Plot of TOF [TOF (h^−1^) = moles of product/moles of catalyst/time (h)] vs. α for **1(TME)**OTf in pseudo-catalytic conditions (Table 2). See text for details.

In order to be able to establish in greater detail the role of solvent during catalysis, the trend of α against TOF [54,55] has been analyzed, which is shown in Figure 4 (data are also present in Table 2). 

From the analysis of Figure 4, it can be seen that the p-cymene, in which **1(TME)**OTf has the highest TOF value of 500 h^−1^, is also the one that has the lowest α of 0, i.e., the highest amount of ion pairs and higher aggregates. The templating and activating effects of OTf^−^ during the nucleophilic attack through the formation of a hydrogen bond thus appear to be increased when aggregates greater than the ion pair (quadruple ions) are present in the catalytic solution. A similar positive effect in the catalysis of aggregations above the ion pair has also been recently observed in metallocenium catalysts for polyolefin synthesis [76].

Also, in Figure 4, it can be seen that chloroform, methanol, acetone, and γ-valerolactone have comparable TOFs (values between 280 h^−1^ and 340 h^−1^) [54,55] despite the significant differences of α values (from 0.24 for chloroform to 0.84 for γ-valerolactone). As noted earlier, the high TOF value in chloroform is related to the low α value, while the high TOF combined with the high α values for methanol, acetone, and γ-valerolactone can be explained through a direct role of the solvent during the nucleophilic attack of methanol.

The higher TOF value calculated for the reaction run in methanol, with respect to that obtained for the reaction run in chloroform, is a direct result of the specific templating and activating roles of other surrounding molecules of MeOH in the reaction mechanism, as predicted by the DFT calculations [57]. Furthermore, the coordination ability of MeOH towards the gold center should be lower than that shown by 3-hexyne [48].

The higher TOF value calculated for the reaction run in acetone and γ-valerolactone, with respect to that obtained for the reaction run in chloroform, can be attributed to a specific character of the carbonyl functionality in the reaction mechanism because the O-H bond may be polarized via specific intramolecular interactions, thus suppressing the anion effect. Both solvents possess functionalities that resemble those present in the DMF and DMPU utilized as appropriate ionic neutral additives in order to increase the rate of the catalytic hydration and alkoxylation of the alkyne reactions [77,78].

These solvents play a fundamental role during the reaction. They do not interact with the NHC-Au^+^ fragment, allowing the coordination of 3-hexyne, and can interact with the MeOH molecule during the nucleophilic attack. These experimental results confirm our DFT calculations. For the more polar γ-valerolactone, the degree of ion-pair separation is superior, and thermodynamically more stable species, both as a reactant complex and transition state, are formed. The transition state stability is additionally enhanced by the reduced (increased) anion affinity for the cationic fragment (the solvent) in the more polar solvent, which in turn increases the electrophilic character of the substrate. Conversely, when free ions are taken into account, cationic transition states are stabilized by more polar γ-valerolactone, which also induces more polarization of the hydroxyl group of methanol [54,55].

## 3. Materials and Methods

### 3.1. Synthesis and Intramolecular Characterization

TME, silver triflate (AgOTf), silver p-toluenesulfonate (AgOTs), silver tetrafluoroborate (AgBF4), and AgBARF were purchased from Sigma Aldrich. All the solvents were used as received without any further purification unless otherwise stated. **1**Cl, **1**OTf, and **1**OTs were synthesized according to the literature [49]. **1(TME)**BF4 and **1(TME)**BARF were generated in situ by adding the appropriate silver salt to a solution of **1**Cl and TME. All compounds were characterized in solution by ^1^H, ^13^C, and ^19^F NMR spectroscopies. The NMR spectra were recorded on an Avance 400 III HD spectrometer. Chemical shifts (ppm) were relative to TMS for both ^1^H and ^13^C nuclei, whereas the ^31^P, ^19^F, and ^15^N chemical shifts were referenced to 85% H_3_PO_4_, CCl_3_F, and CH_3_NO_2_, respectively. The complexes were fully characterized with mono-dimensional (^1^H and ^13^C) and bi-dimensional (^1^H-^1^H COSY, ^1^H-^1^H NOESY, ^1^H-^13^C HSQC, ^1^H-^13^C HMBC) NMR experiments. All the experimental details and NMR data are reported in the Supporting Information. 

### 3.2. DOSY Measurements

The DOSY NMR spectra were acquired using a Bruker Avance III HD 400 MHz spectra equipped with a broadband 5 mm probe (^1^H/BBF iProbe) with a *z*-axis gradient (50 G/cm) at 298 K without sample spinning. The DOSY experiments were carried out using the double-stimulated echo version with a longitudinal eddy current delay (*dstegp3s* sequence). The gradient pulse (P30, δ) was set to 1750 us, while the diffusion time (D20, Δ) was set to 0.1 s, and the eddy current delay (D21) was set to 5 ms. The experiments were acquired using the “dosy” AU program, collecting a total of 32 points (TD1 entry) following a linear ramp with a gradient intensity (g) ranging from 95% to 5% (47.187 dB to 0.963 dB). The number of scans (ns) was set to 64. 

**Figure 5:**
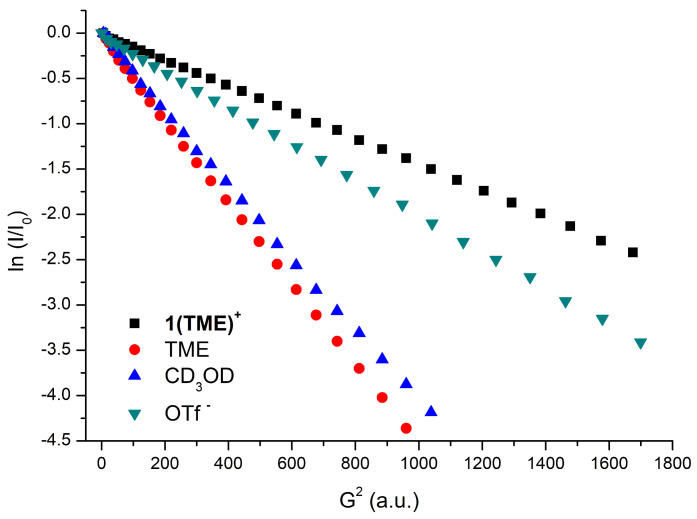
Plot of ln(I/I0) vs. G^2^ for complex **1(TME)**OTf in CD_3_OD (Table 2, entry 3).

The dependence of the resonance intensity (I) on a constant waiting time and on a varied gradient strength G is described by the following Equation (4):

(4) $$\ln\frac{I}{I_{0}}=-(\gamma \delta {)}^{2}D_{t}\left(\Delta -\frac{\delta }{3}\right)G^{2}$$

where I is the intensity of the observed spin echo, I_0_ is the intensity of the spin echo in the absence of a gradient, D_t_ is the self-diffusion coefficient, and Δ is the delay between the midpoints of the gradients, δ, the length of the gradient pulse, and γ the magnetogyric ratio. The semilogarithmic plots of ln(I/I_0_) versus G^2^ were fitted by using a standard linear regression algorithm, and a correlation factor better than 0.99 was always obtained (Figure 5). The self-diffusion coefficient D_t_, which is directly proportional to the slope m of the regression line obtained by plotting ln(I/I_0_) versus G^2^, was estimated by evaluating the proportionality constant for a sample of HDO (5%) in D_2_O (known diffusion coefficients in the range 274–318 K) [38] under the exact same conditions as the sample of interest. The solvent (or 2,3-dimethyl-2-butene, TME) was taken as an internal standard. The D_t_ data were treated as described in the literature to derive the hydrodynamic dimensions. 

## 4. Conclusions

Our research demonstrates that the combination of DOSY NMR diffusion experiments and kinetic measurements of gold catalyst activity is a reliable methodology for understanding the behavior of gold catalysts for the alkoxylation of unsaturated hydrocarbon and disclosing the role of ion pairs (the amount of free ions, ion pairs, and higher aggregates), the nature of anions (coordination ability, basicity, and hydrogen bond acceptor properties), and solvent (polarity, gold affinity, and the hydrogen-bond basicity of peculiar functional groups).

We have successfully applied this methodology to the investigation of gold complexes [(NHC)AuX] and [(NHC)Au(TME)X] [NHC 1,3-bis(2,6-di-isopropylphenyl)-imidazol-2-ylidene; TME = tetramethylethylene (2,3-bis methyl-butene)], which change in the nature of the counterion [Cl^−^, BF_4_^−^, OTf^−^; OTs^−^ BArF_4_^−^ (ArF = 3,5-(CF_3_)_2_C_6_H_3_)] in different catalytic solvents (p-cymene, chloroform, methanol, acetone, γ-valerolactone, and propylene carbonate).

The ^1^H and ^19^F DOSY NMR measurements conducted in catalytic conditions in chloroform indicate that α (quantity of free ions) depends on the nature of the counterion. While the compounds containing OTs^−^ and OTf^−^ as counterions gave a low α, the catalysts containing BF_4_^−^ and BArF_4_^−^ recorded a higher α. Analyzing the trend of α against TOF (Figure 3), it can be seen that the TOF is inversely proportional to α. This is because the anion helps with a templating effect during the nucleophilic attack of methanol, which is only possible with ion pairs and absent when the catalyst is present in the solution in the form of free ions. However, the difference in reactivity between **1(TME)**OTs and **1(TME)**OTf is likely due to a higher templating power of OTs^−^ with respect to OTf^−^, as suggested by previous DFT calculations [58].

Furthermore, α increases as the dielectric constant increases, as expected. However, there are additional considerations that can be made when α data are considered in conjunction with TOF(Figure 4). The DOSY measurements conducted in p-cymene show the formation of quadrupole species. These conditions allow the anion to better exercise its ‘template’ and ‘activating’ roles, resulting in higher TOFs than in chloroform. Chloroform and γ-valerolactone have comparable TOFs (Figure 4) despite there being significant differences in the α. A high TOF, combined with the high α values for γ-valerolactone, experimentally confirms the direct role of the solvent during the nucleophilic attack of methanol, as previously suggested by the DFT calculations [54,55].

In conclusion, for the first time in the literature, we focused on gold catalysis. The combination of experimental (and theoretical) catalytic data and NMR diffusion results allows us to analyze the outcomes in-depth and quantitatively as follows: (1) the role of the anion during the nucleophilic attack (Figure 1) disentangles the ion-pairing and different templating and activating effects of the counterion (hydrogen bond acceptor property), and (2) the role of the solvent during gold catalysis disentangles the polarity effect (related to ion pairing) and activating effect due to the presence of specific functionalities (hydrogen-bond basicity).

## Figures and Tables

**Table 1:** Diffusion coefficient (10^−10^ m^2^s^−1^), hydrodynamic radius (r_H_^±^, Å), hydrodynamic volume (V_H_^±^, Å^3^), aggregation number (N^±^), and α for complexes **1**Cl and **1(TME)**X (in parenthesis, the V_H_^0,IP^ and Å^3^; see text for details) in pseudo-catalytic conditions (400 μL of CDCl_3_, 142 μL of CD_3_OD, and 105 μL of TME).

Entry	Compound	D_t_^+^	D_t_^−^	r_H+_	V_H_^+^	r_H_^−^	V_H_^−^	N^+^	N^−^	α
1	**1**Cl (937)	6.99		6.1	937			1.00		
2	**1(TME)**OTs (1245)	5.93	5.24	6.6	1188	6.4	1098	0.95	0.88	0.13
3	**1(TME)**OTf (1152)	6.40	7.38	6.2	998	6.0	900	0.87	0.78	0.24
4	**1(TME)**BF_4_ (1089)	6.01	7.65	6.9	1376	5.6	720	1.26	0.66	0.36
5	**1(TME)** BARF (1927)	6.84	5.58	6.7	1260	7.3	1596	0.65	0.82	0.32

**Table 2:** Diffusion coefficient (10^−10^ m^2^s^−1^), hydrodynamic radius (r_H_^±^, Å), hydrodynamic volume (V_H_^±^, Å^3^), aggregation number (N^±^), and α for complexes **1(TME)**OTf (V_H_^0,IP^ = 1152 Å^3^; see text for details) in pseudo-catalytic conditions in different solvents (400 μL of solvent, 142 μL of CD_3_OD, and 105 μL of TME).

Entries	Solvent	D_t_^+^	D_t_^−^	r_H_^+^	V_H_^+^	r_H_^−^	V_H_^−^	N^+^	N^−^	α
1	p-cymene	3.60	5.26	8.4	2509	7.1	1499	2.18	1.3	0
2	chloroform *	6.40	7.38	6.2	998	6.0	900	0.87	0.78	0.24
3	methanol	6.57	10.5	6.5	1166	4.2	306	1.01	0.26	0.70
4	acetone	14.6	10.2	6.1	946	3.9	250	0.82	0.22	0.80
5	γ-valerolactone	3.52	7.94	6.3	1067	4.0	270	0.93	0.23	0.84
6	propylene carbonate	3.56	9.47	6.3	1023	3.7	217	0.89	0.18	0.90

* entry 3 Table 1, for comparison.

## Data Availability

All the data are present in the Supporting Information.

## Supplementary Materials

The following supporting information can be downloaded at: https://www.mdpi.com/article/10.3390/molecules29133018/s1, Experimental details of the synthesis of gold compounds, DOSY measurements original data, Tables S1–S4 and Figures S1–S28; References [79,80,81,82,83,84,85,86,87,88].
