# Supplementary material for: Diffusion Nuclear Magnetic Resonance Measurements on Cationic Gold (I) Complexes in Catalytic Conditions: Counterion and Solvent Effects
Source: Molecules. 2024 Jun 26;29(13):3018. doi: 10.3390/molecules29133018 (PMC11243661; doi:10.3390/molecules29133018)
Supplement: Supplementary file 1 [file molecules-29-03018-s001.zip › molecules-3002604-supplementary.pdf]

Diffusion Nuclear Magnetic Resonance Measurements on Cationic Gold (I) Complexes in Catalytic Conditions: Counterion and Solvent Effects

Filippo Campagnolo,[a] Eleonora Aneggi,[a] Walter Baratta,[a] Talha Munir, [a] Daniele Zuccaccia,[a]*

[a] Dipartimento di Scienze Agroalimentari, Ambientali e Animali, Sezione di Chimica, Università di Udine, Via Cotonificio 108, I-33100 Udine, Italy. E-mail: daniele.zuccaccia@uniud.it

* Correspondence: (DZ) daniele.zuccaccia@uniud.it

Table of contents:

1 Materials and apparatus 3

2 Synthesis of chloro[1,3-bis(2',4',6'-diisopropylphenyl)imidazol-2-ylidene]gold(I) (1Cl): 4

2.1 Procedure: .... **Error! Bookmark not defined.**

2.2 Characterization: .... **Error! Bookmark not defined.**

3 Synthesis of p-toluenesulfonate [1,3-bis(2',4',6'-diisopropylphenyl)imidazol-2-ylidene]gold(I) (1OTs): 5

3.1 Procedure: 5

3.2 Characterization: .... **Error! Bookmark not defined.**

4 Synthesis of trifluoromethanesulfonate [1,3-bis(2',4',6'-diisopropylphenyl)imidazol-2-ylidene]gold(I) (1OTf): 6

4.1 Procedure: 6

4.2 Characterization: .... **Error! Bookmark not defined.**

5 DOSY NMR measurements 7

5.1 Determination of the average hydrodynamic volume (V_H) following DOSY methodology 7

5.2 Standard DOSY experiment parameters (for Topspin v3.6.2): 10

5.3 Determination of internal standards' $c(r_{solv}, r_H)*r_H$: 10

5.4 A note on the naming of chemical species: .... **Error! Bookmark not defined.**

5.5 Consistency between ^{1}H-DOSY and ^{19}F-DOSY NMR .... **Error! Bookmark not defined.**

5.6 Determination of catalysts' D_t in pure chloroform: 12

5.7 Pseudo-catalysis experiment for pre-activated catalysts: 20

5.8 Pseudo-catalysis experiment involving *in situ* catalyst activation: 24

5.9 Pseudo-catalysis experiment involving different solvents: 28

6 Data analysis 39

6.1 Calculated hydrodynamic volumes in pure chloroform 39

7 References: 41

1 Materials and apparatus

- All reactants have been purchased from Merk® (formerly Sigma-Aldrich®) and used as delivered if not otherwise stated.
- The NMR spectra were acquired using a Bruker Avance III HD 400MHz spectra equipped with a broadband 5mm probe (^1H/BBF iProbe) with a z-axis gradient (50G/cm).

2 Synthesis of chloro[1,3-bis(2',4',6'-diisopropylphenyl)imidazol-2-ylidene]gold(I) (1Cl):

A literature-available synthetic protocol was followed with minor variations.[1] A round bottom flask was charged with 300 mg (0.9 mmol, 1.0 eq) of *chloro(tetrahydrothiophene)gold(I)*, 374 mg (3.6 mmol, 4 eq) of *potassium hydrogen carbonate*, and 417 mg (0.9 mmol, 1.0 eq) of *1,3-Bis(2,6-diisopropyl phenyl)imidazolium chloride*. The solids were dissolved using 25 mL of a 5:1 volume ratio of a *DCM-methanol* solution. After 24 hours, the mixture was filtered over Celite, and was washed with 2 x 5 mL *DCM* aliquots. The filtrate was reduced in volume and cooled to 0 °C, then *pentane* was added under vigorous stirring resulting in product precipitation. The white solid was isolated via Gooch filtration, washed with *pentane* (2 x 5 mL), and dried under vacuum (yield: 90%).

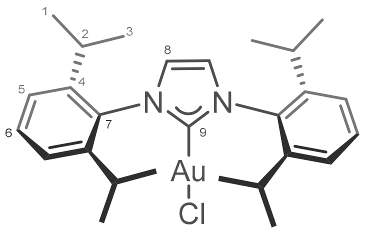

^{1}H-NMR($CDCl_3$, 400 MHz) δ(ppm): 7.52 (t, 2H, $^{3}J_{H6\text{-}H5}$ = 7.81 Hz, **6**), 7.31 (d, 4H , $^{3}J_{H5\text{-}H6}$ = 7.82 Hz, **5**), 7.19 (s, 2H, **8**), 2.58 (hept, 4H, $^{3}J_{H2\text{-}H1\ /H2\text{-}H3}$ = 6.83 Hz, **2**), 1.37 (d, 12H, $^{3}J_{H1\text{-}H2}$ = 6.88 Hz, **3**), 1.24 (d, 12H, , $^{3}J_{H2\text{-}H3}$ = 6.88 Hz, **1**). ^{13}C-NMR (CDCl3): δ (ppm) 175.42 (**9**), 145.61 (**4**), 134.01 (**7**), 130.77 (**6**), 124.32 (**5**), 123.03 (**8**), 28.84 (**2**)**,** 24.50 (**1**), 24.04 (**3**)

3 Synthesis of p-toluenesulfonate [1,3-bis(2’,4’,6’-diisopropylphenyl)imidazol-2-ylidene]gold(I) (1OTs):

3.1 Procedure:

A literature-available synthetic protocol was followed with minor variations.[1] A round bottom flask was charged with 150 mg (0.24 mmol, 1.0 eq) of *chloro[1,3-bis(2',4',6’-trimethylphenyl)imidazol-2-ylidene]gold(I)* and 74 mg (0.27 mmol, 1.1 eq) of *silver p-toluenesulfonate*, than 10 ml of a 5:1 ratio *DCM* and *methanol* solution were added. The mixture reacted for 24 h shielded by light and was subsequently filtered over Celite pad, which was washed with 2 x 2.5 mL *DCM* aliquots. The filtrate was reduced in volume and cooled to 0°C, and *pentane* was added under vigorous stirring. The white precipitate was isolated via gooch filtration, washed with *pentane* (2 x 5 mL), and dried under vacuum (yield: 75%).

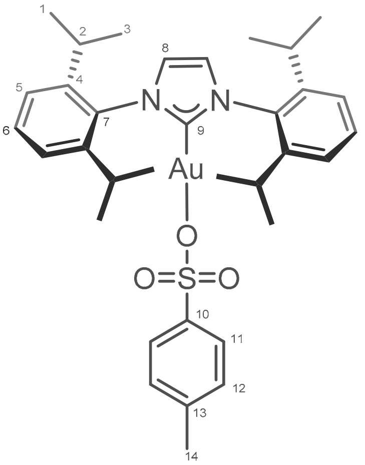

^{1}H-NMR($CDCl_3$, 400 MHz) δ(ppm): 7.57 (t, 2H, $^{3}J_{H6-H5}$ = 7.81 Hz, **6**), 7.42 (d, 2H, $^{3}J_{H11-H12}$ = 7.41 Hz, **11**), 7.33 (d, 4H , $^{3}J_{H5-H6}$ = 7.32 Hz, **5**), 7.23 (s, 2H, **8**), 7.00 (d, $^{3}J_{H12-H11}$ =7.91 Hz, 2H), 2.51 (hept, 4H, $^{3}J_{H2-H1\,/H2-H3}$ = 7.05 Hz, **2**), 2.35 (s, 3H, **14**), 1.31 (d, 12H, $^{3}J_{H1-H2}$ = 7.25 Hz, **3**), 1.24 (d, 12H, $^{3}J_{H3-H2}$ = 6.88 Hz, **1**)

4 Synthesis of trifluoromethanesulfonate [1,3-bis(2',4',6'-diisopropylphenyl)imidazol-2-ylidene]gold(I) (1OTf):

4.1 Procedure:

A literature-available synthetic protocol was followed with minor variations.[1] A round bottom flask was charged with 150 mg (0.24 mmol, 1.0 eq) of *chloro[1,3-bis(2',4',6'-trimethylphenyl)imidazol-2-ylidene]gold(I)* and 74 mg (0.27 mmol, 1.1 eq) of *silver trifluoromethanesulfonate*, than 10 ml of a 5:1 ratio *DCM* and *methanol* solution were added. The mixture reacted for 24 h shielded by light and was subsequently filtered over Celite pad, which was washed with 2 x 2.5 mL *DCM* aliquots. The filtrate was reduced in volume and cooled to 0°C, and *pentane* was added under vigorous stirring. The white precipitate was isolated via gooch filtration, washed with *pentane* (2 x 5 mL), and dried under vacuum (yield: 85%).

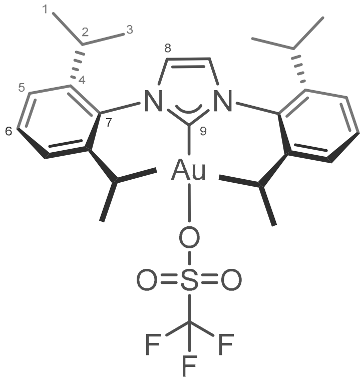

^{1}H-NMR($CDCl_3$, 400 MHz) δ(ppm): 7.57 (t, 2H, $^3J_{H6-H5}$ = 7.68 Hz, **6**), 7.34 (d, 4H, $^3J_{H5-H6}$ = 7.68 Hz, **5**), 7.28 (s, 2H, **8**), 2.51 (hept, 4H, $^3J_{H2-H1\ /H2-H3}$ = 7.13 Hz, **2**), 1.34 (d, 12H, $^3J_{H1-H2}$ = 7.13 Hz, **3**), 1.25 (d, 12H, $^3J_{H3-H2}$ = 7.43 Hz, **1**)
^{19}F-NMR($CDCl_3$, MHz) δ(ppm): -77.19 (s, 3F)

5 DOSY NMR measurements

5.1 Determination of the average hydrodynamic volume (V_H) following DOSY methodology

DOSY NMR experiments allow for determining the translational diffusion coefficients (Dt) of chemical species within a sample under specific conditions.[2,3] In fact, when a pulsed frequency gradient (PFG) is applied to a sample, the signal intensity of each species is reduced in function to its hydrodynamic volume, a phenomenon described by the Stejskal-Tanner equation:[4]

$$E_{Diff} = e^{-\gamma^2 \delta^2 g^2 D_t \sigma \Delta'}$$

Equation S1 – General form of the Stejskal-Tanner equation, where D_t is the translational diffusion coefficient, γ is a linear combination of the gyromagnetic ratios of the nuclei studied, δ is the pulsed frequency gradient (PFG) duration, σ is the gradient shape factor, Δ is the time during which the molecular diffusion induces its effects, while Δ' is the same term corrected to account for shape factors and finally, g represent the gradient strength.

While the general form of the Stejskal-Tanner equation presented above is correct (Equation S1), in practice, it needs to be adjusted to account for various factors dependent on the PFG shape and the specific sequence employed.[5] In our study, we employed the *dstegp3s* sequence developed by Jerschow et al., a double-stimulated echo sequence designed to address convective motion effects, thus requiring a slight modification of the original general form:[5–7]

$$E_{Diff} = e^{-\gamma^2 \delta^2 g^2 D_t [\Delta + 4k - 4\lambda)]} = e^{-\gamma^2 \delta^2 g^2 D_t \left[\Delta - \frac{2}{3}\right]}$$

Equation S2 – The Stejskal-Tanner equation adapted for the sequence used in the present work, the right side of the equation has been adapted to account for a rectangular-shaped gradient, with σ = 1, λ = ½, and k = ⅓.

Consequently, after conducting a DOSY experiment, the D_t values for the relevant compounds in the sample can be obtained by fitting Equation S2 on a plot displaying the normalized signal intensity (I/I_0) of a species against gradient strength (g). The range of g values must be sufficient to cause proper signal attenuation to achieve a reliable fitting. Alternatively, and more conveniently, a D_t value can be determined by exploiting its proportionality to the slope of the regression line obtained by plotting the logarithm of the normalized signal intensity ($\log(I/I_0)$) against the square of the pulsed frequency gradient strength (g^2). However, determining the proportionality factor is crucial for obtaining meaningful D_t values, and this can be achieved by conducting measurements on a sample of D_2O containing 5% HDO, for which the D_t values are known across a range of temperatures.[8,9]

From D_t of a chemical species, the average hydrodynamic radius (r_H) and hydrodynamic volume (V_H) can be calculated via the Stokes-Einstein equation:

$$D_t = \frac{kT}{6\pi\eta r_H} = \frac{kT}{c(r_{solv}, r_H) f_s(a,b)\pi\eta r_H} \rightarrow V_H = \frac{4}{3}\pi r_H^3$$

Equation S3 – On the left side, the original Stokes-Einstein equation where k represents Boltzmann's constant, T the temperature, η the medium's viscosity. On the right side, a modified version of the Stokes-Einstein equation where $c(r_{solv}, r_H)$ and fs(a,b) are the coefficients accounting for a particle's dimension relative to the media and the particles' shape. Finally, the volume of a given spherical particle is calculated from the r_H.

While the Stokes-Einstein equation is designed to describe a spherical particle whose dimensions are orders of magnitude compared to the media, these assumptions are not always valid at the molecular level. For these reasons, the determination of r_H from D_t is not trivial.[10] For instance, when the chemical species under study are roughly similar in size to the solvent, the medium is no longer a continuum. In such cases, solvation effects and the shape of the particles could impact diffusion, causing a deviation from the behavior predicted by the original Stokes-Einstein equation. In this study, the shape of the particles is assumed to be spherical,[11] thus the $f_s(a,b)$ parameter is always equal to one. The numerical factor $c(r_{solv}, r_H)$ is instead estimated following a semi-empirical approach developed by Chen et al.:[12]

$$c(r_{solv}, r_H) = \frac{6}{1 + 0.695\left(\frac{r_{solv}}{r_H}\right)^{2.234}}$$

Equation S4 – The semi-empirical relationship developed by Chen et al. to estimate the value of $c(r_{solv}, r_H)$.[12]

Conveniently, the η and T terms in Equation S3 do not need to be evaluated, provided that an internal standard is introduced in the sample. The introduction of an internal standard also corrects possible errors in the calibration of the PFG.[13] However, for an internal standard to be reliable, it must meet specific criteria. It should possess at least one resonance falling within a free region of the NMR spectrum, be soluble in commonly used solvents, be thermally stable, chemically inert, and should not self-aggregate. Furthermore, the introduction of a reliable internal standard significantly simplifies the determination of D_t for unknown species. Conducting a DOSY measurement with an internal standard while carefully controlling the experimental conditions, such as η, T, and g, allows for the determination of D_t and, consequently, the $c(r_{solv}, r_H)f_s(a,b)r_H$ product. Assuming spherical chemical species, the equation can be easily solved for r_H using the semi-empirical relationship developed by Chen et al. (Equation S4).

Most importantly, since the D_t values of the internal standard and an analyte can be determined in the same measurement, the $c(r_{solv}, r_H)*r_H$ value of the analyte can be obtained by combining Equation S3 for both the internal standard and the analyte.[10,13]

$$\frac{D_t^{sa}}{D_t^{st}} = \frac{m^{sa}}{m^{st}} = \frac{c(r_{solv}, {r_H}^{st}) f_s(a,b)^{st} r_H^{st}}{c(r_{solv}, {r_H}^{sa}) f_s(a,b)^{st} r_H^{sa}} = \frac{c(r_{solv}, {r_H}^{st}) r_H^{st}}{c(r_{solv}, {r_H}^{sa}) r_H^{sa}}$$

Equation S5 – D_t ratio of the sample against the internal standard. It can be noted that the ratio of the D_t values is not affected by η, T, and g. Moreover, since the D_t values are proportional to the slope obtained by plotting the logarithm of the normalized signal intensity ($\log(I/I_0)$) against the square of the pulsed frequency gradient strength (g^2), by proportionality, D_t values are not required to be determined, as the D_t ratio exactly corresponds to the ratio of the slopes.

Given that $c(r_{solv}, r_H)^{st}*r_H^{st}$ / $c(r_{solv}, r_H)^{sa}*r_H^{sa}$ can be determined from the obtained D_t ratio, with $c(r_{solv}, r_H)^{st}*r_H^{st}$ known from prior measurements, $c(r_{solv}, r_H)^{sa}*r_H^{sa}$ can be subsequently calculated. Followingly, r_H^{sa} can finally be determined by graphically solving the dependence of $c(r_{solv}, r_H)*r_H$ on r_H (Figure).[13]

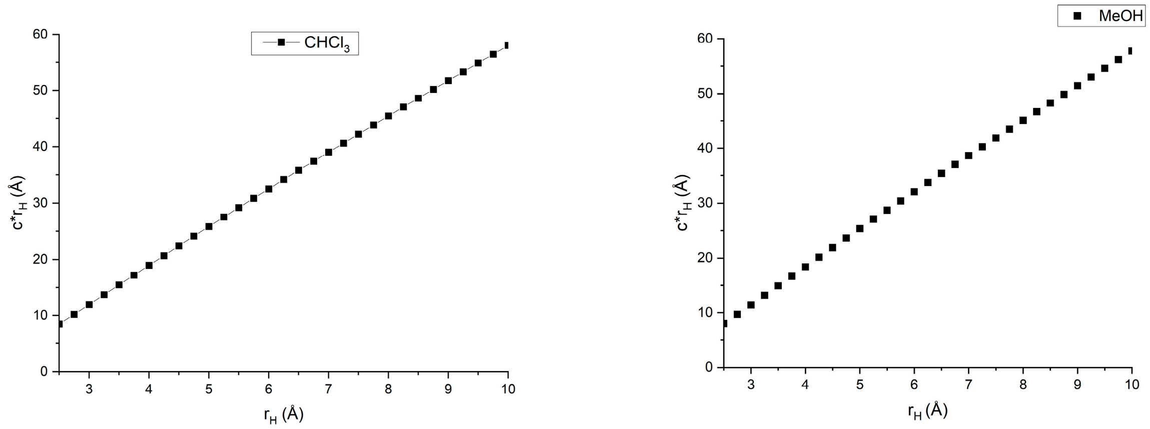

Figure S1 – The reported graphs display $c(r_{solv}, r_H)\cdot r_H$ as a function of r_H following the semi-empirical relationship developed by Chen et al.,[12] for a chemical species diffusing in *chloroform* (left) and *methanol* (right).[12]

5.2 Standard DOSY experiment parameters (for Topspin v3.6.2):

The DOSY NMR spectra were acquired using a Bruker Avance III HD 400MHz spectra equipped with a broadband 5mm probe (^{1}H/BBF iProbe) with a z-axis gradient (50 G/cm) at 298 K without sample spinning. The DOSY experiments were carried out using the *dstegp3s* sequence. The gradient pulse (P30, δ) was set to 1750 us, while the diffusion time (D20, Δ) was set to 0.1 s, and the eddy current delay (D21) was set to 5 ms. The experiments were acquired using the "dosy" AU program, collecting a total of 32 points (TD1 entry) following a linear ramp with gradient intensity (g) ranging from 95% to 5% (47.187 dB to 0.963 dB). The number of scans (ns) was set to 64.

5.3 Determination of internal standards' $c(r_{solv}, r_H)*r_H$:

Under catalytic conditions, *tetramethylethylene (2,3-dimethyl-2-butene or TME)* was employed as an internal standard for two reasons: 1) the *chloroform* signal sometimes overlaps with critical signals of other chemical species, and 2) the steep decay of *chloroform's* signal intensity affords fewer points to perform a suitable linear regression. The $c(r_{solv}, r_H)*r_H$ of *tetramethylethylene* was determined in *chloroform* using $c(r_{solv}, r_H)*r_H$ of *chloroform* itself as an internal standard, finding consistent values (Figure XX, Table 1).

Table S1 - Calculated hydrodynamic volumes (V_H) of *tetramethylethylene* as determined by ^{1}H DOSY NMR spectroscopy according to the disclosed procedure and using $CDCl_3$ as internal standard.

Compound	D_t	Error	m	r_H	V_H
[Code]	[m^2/s]	[%]	[u]	[A]	[A^3]
TME	2.52E-09	4.65	-3.99E-03	2.9	97
$CHCl_3$	2.18E-09	3.11	-4.49E-03	2.8	87

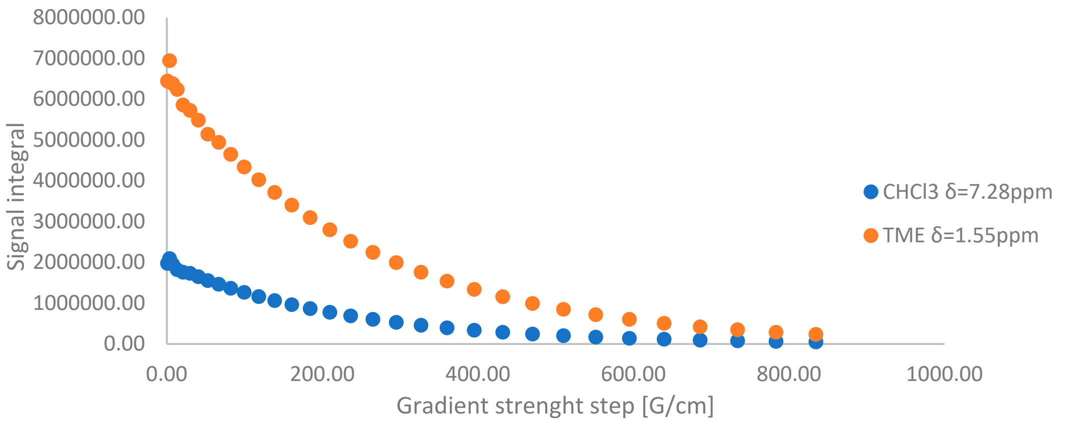

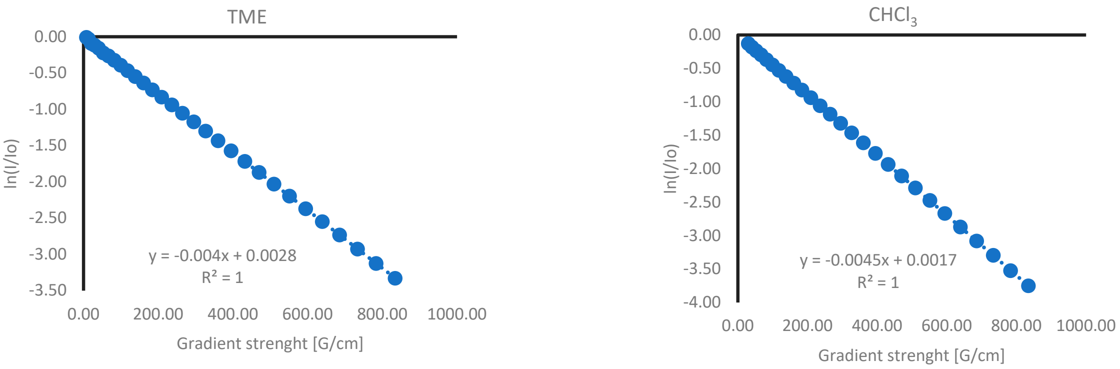

Figure S2 – Signal decay of the relevant chemical species, as observed during the DOSY NMR experiment. The sample contained (100 uL, 1.2 mmol) of *chloroform,* (500 uL, 6.2 mmol) of deuterated *chloroform,* and (10 uL, 0.1 mmol) of *tetramethylethylene.*

5.4 Determination of catalysts' D_t in pure chloroform:

1OTs, 1OTf. A 4 mL vial was charged with $8.8 \cdot 10^{-3}$ mmol (0.01 eq) of the appropriate NHC-gold(I) catalyst. Subsequently, 550 uL (6.85 mmol, 7.78 eq) of deuterated *chloroform* where added to the vessel, which was placed under stirring until a homogeneous solution was formed. Finally, the solution was transferred to a 5 mm NMR tube for analysis.

1(TME)BF_4, 1(TME)BARF. A 4 mL vial was charged with $8.8 \cdot 10^{-3}$ mmol (0.01 eq) of **1Cl**, under argon, with $8.8 \cdot 10^{-3}$ mmol (0.01 eq) of the appropriate silver salt ($AgBF_4$ or AgBARF) and with $4.4 \cdot 10^{-2}$ mmol (0.05 eq) of *tetramethylethylene*. Subsequently, 550 uL (6.85 mmol, 7.78 eq) of deuterated *chloroform* where added to the vessel, which was placed under stirring for 30 minutes. Finally, the solution was filtered and transferred to a 5 mm NMR tube for analysis.

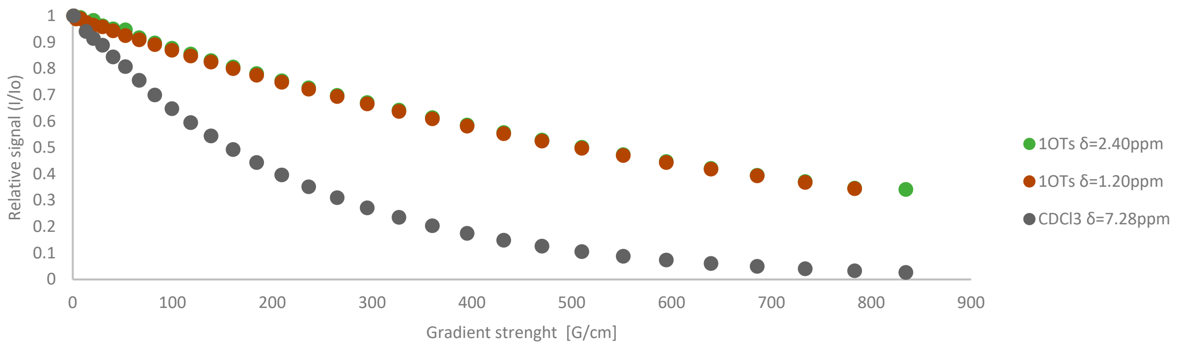

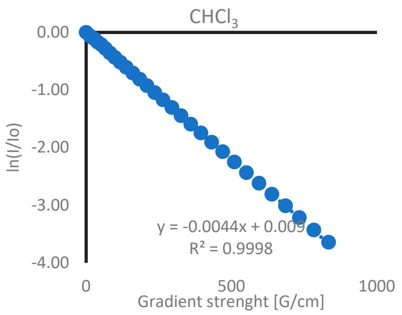

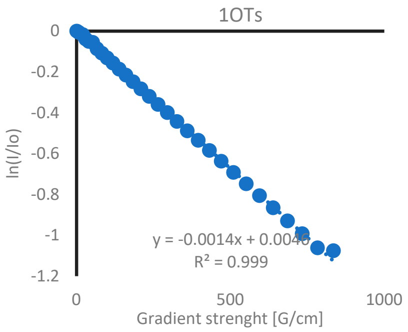

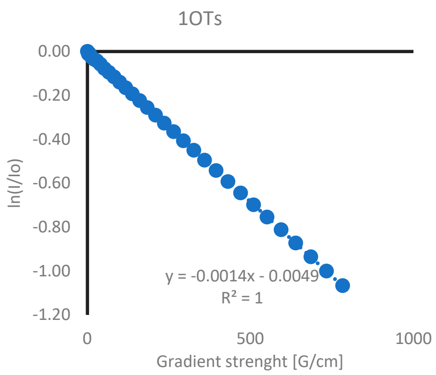

Figure S3 – Signal decay of the relevant chemical species, as observed during the ^{1}H-DOSY NMR experiment. The sample contained 6.46 mg (8.8 · 10^{-3} mmol, 0.01 eq) of *p-toluenesulfonate [1,3-bis(2',4',6'-diisopropylphenyl)imidazol-2-ylidene]gold(I)* (**1OTs**), and 550 uL (6.85 mmol, 7.78 eq) of deuterated *chloroform*.

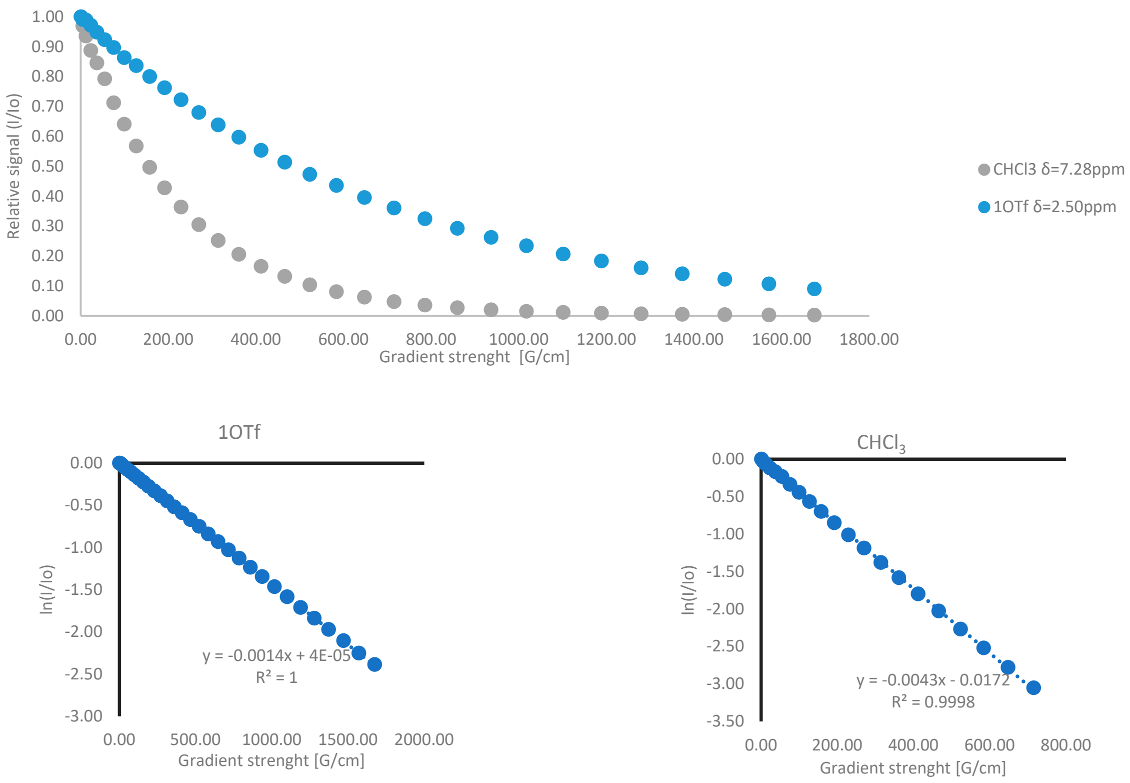

Figure S4 – Signal decay of the relevant chemical species, as observed during the ^{1}H-DOSY NMR experiment. The sample contained 6.46 mg (8.8 · 10^{-3} mmol, 0.01 eq) of *trifluoromethanesulfonate [1,3-bis(2',4',6'-diisopropylphenyl)imidazol-2-ylidene]gold(I)* (**1OTf**), and 550 uL (6.85 mmol, 7.78 eq) of deuterated *chloroform*.

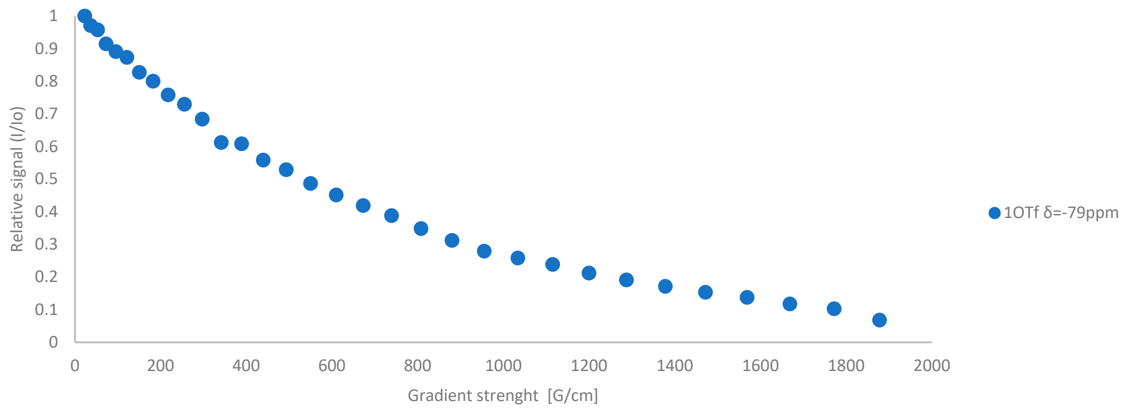

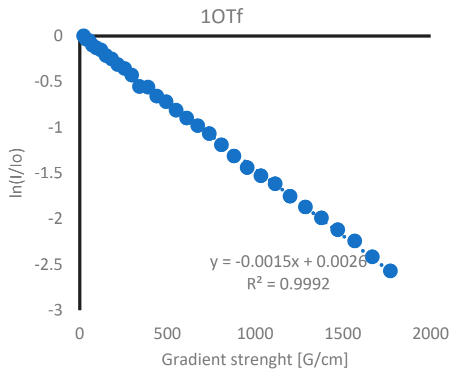

Figure 5 – Signal decay of the relevant chemical species, as observed during the ^{19}F-DOSY NMR experiment. The sample contained 6.46 mg ($8.8 \cdot 10^{-3}$ mmol, 0.01 eq) of *trifluoromethanesulfonate [1,3-bis(2',4',6'-diisopropylphenyl)imidazol-2-ylidene]gold(I)* (**1OTf**), and 550 uL (6.85 mmol, 7.78 eq) of deuterated *chloroform*.

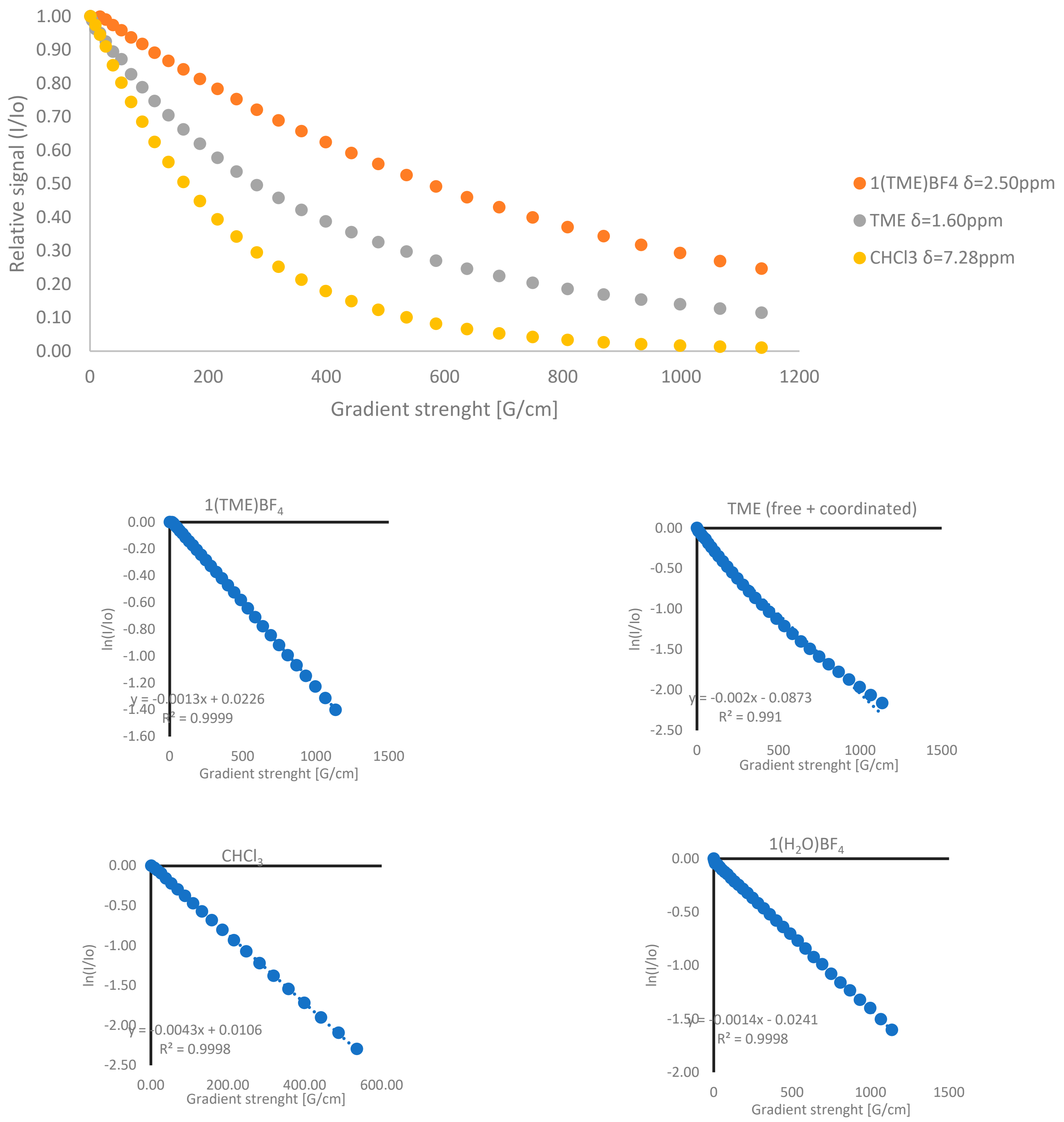

Figure S6 – Signal decay of the relevant chemical species, as observed during the ^{1}H-DOSY NMR experiment. The sample contained 5.46 mg ($8.8 \cdot 10^{-3}$ mmol, 0.01 eq) of *chloro [1,3-bis(2',4',6'-diisopropylphenyl)imidazol-2-ylidene]gold(I)* (**1Cl**), 0.3 mg ($8.8 \cdot 10^{-3}$ mmol, 0.01 eq) of *silver tetrafluoroborate,* $4.4 \cdot 10^{-2}$ mmol (0.05 eq) of *tetramethylethylene* and 550 uL (6.85 mmol, 7.78 eq) of deuterated *chloroform.*

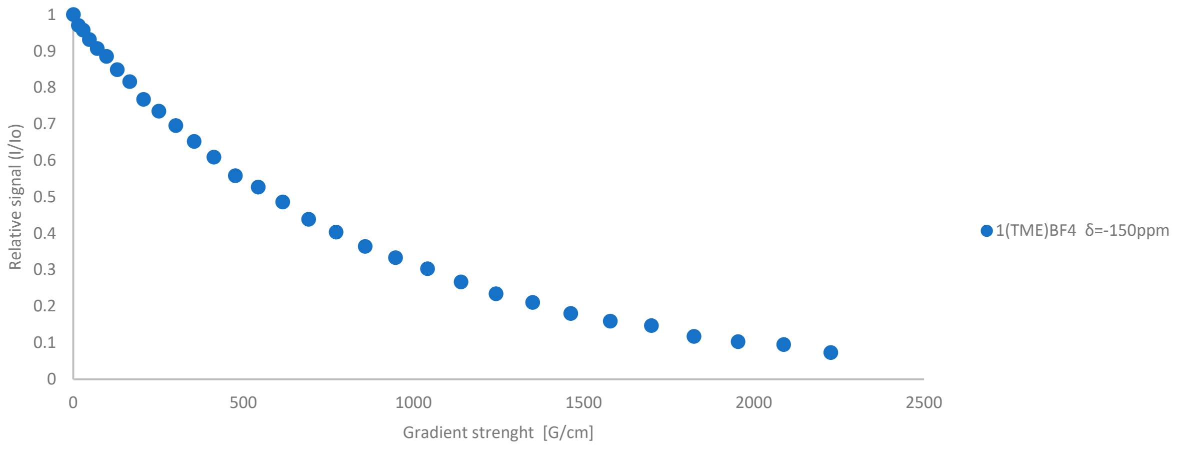

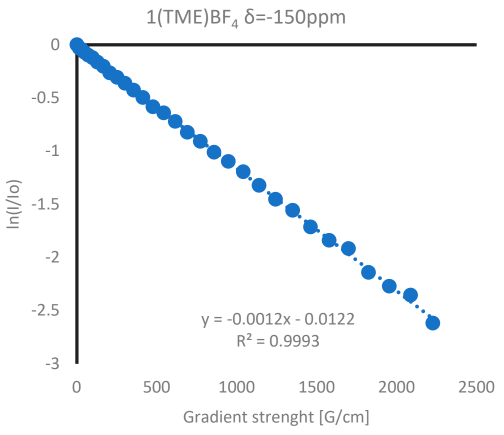

Figure S7 – Signal decay of the relevant chemical species, as observed during the ^{19}F-DOSY NMR experiment. The sample contained 5.46 mg ($8.8 \cdot 10^{-3}$ mmol, 0.01 eq) of *chloro [1,3-bis(2',4',6'-diisopropylphenyl)imidazol-2-ylidene]gold(I)* (**1Cl**), 0.3 mg ($8.8 \cdot 10^{-3}$ mmol, 0.01 eq) of *silver tetrafluoroborate,* $4.4 \cdot 10^{-2}$ mmol (0.05 eq) of *tetramethylethylene* and 550 uL (6.85 mmol, 7.78 eq) of deuterated *chloroform.*

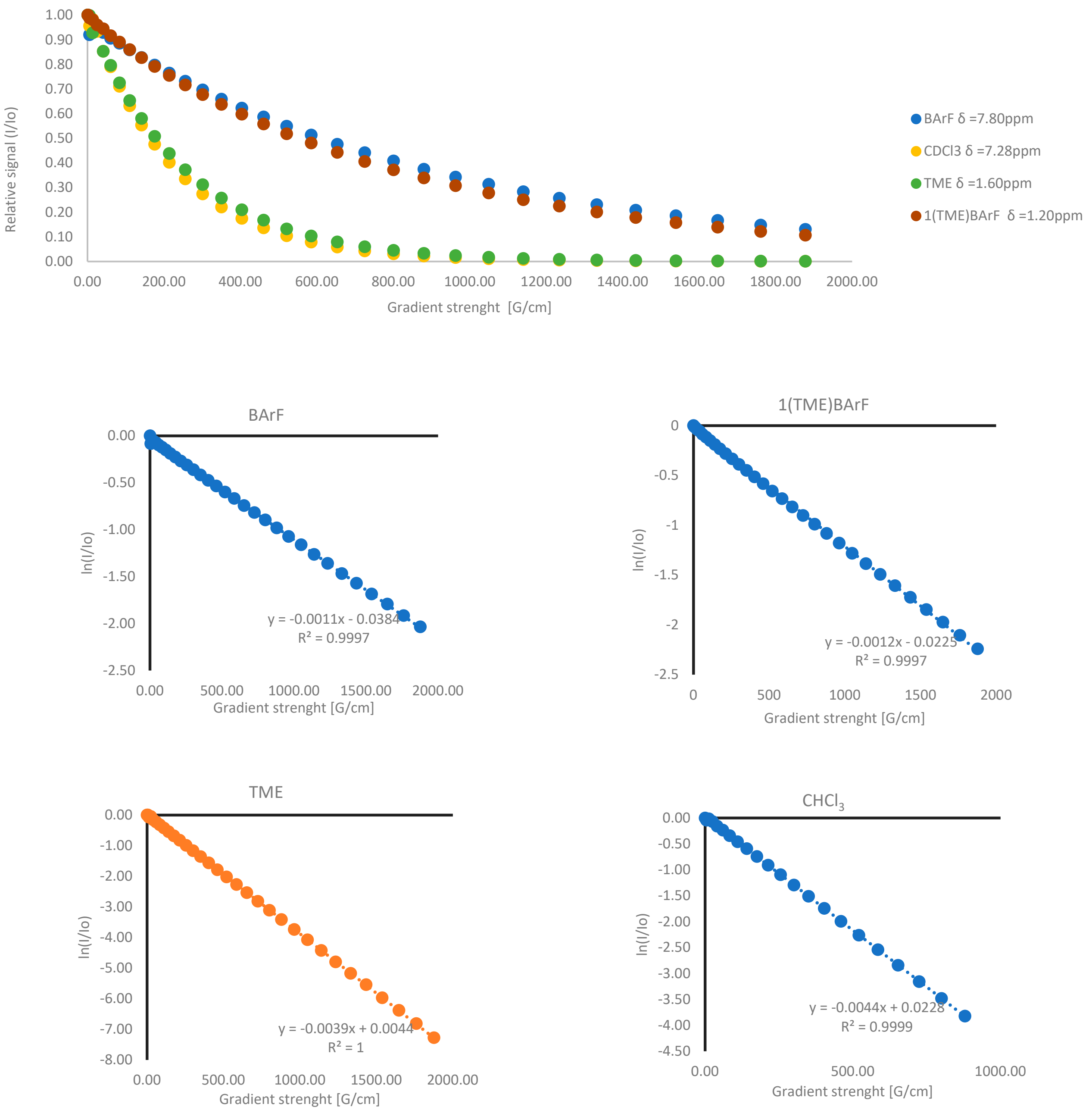

Figure S8 – Signal decay of the relevant chemical species, as observed during the ^{1}H-DOSY NMR experiment. The sample contained 5.46 mg (8.8 · 10^{-3} mmol, 0.01 eq) of *chloro [1,3-bis(2',4',6'-diisopropylphenyl)imidazol-2-ylidene]gold(I)* (**1Cl**), 0.8 mg (8.8·10^{-3} mmol, 0.01 eq) of *silver tetrakis(3,5-bis(trifluoromethyl)phenyl)borate*, 4.4·10^{-2} mmol (0.05 eq) of *tetramethylethylene* and 550 uL (6.85 mmol, 7.78 eq) of deuterated *chloroform*.

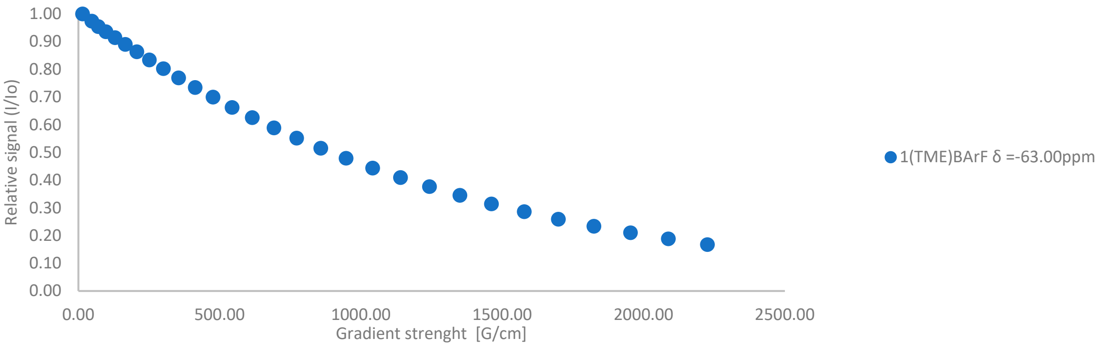

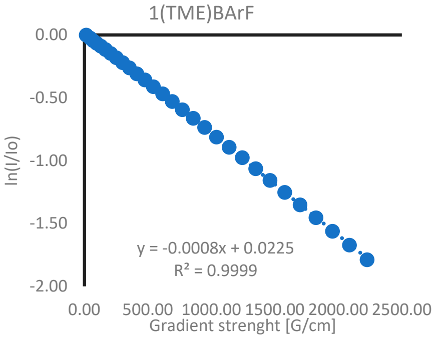

Figure S9 – Signal decay of the relevant chemical species, as observed during the ^{19}F-DOSY NMR experiment. The sample contained 5.46 mg (8.8 · 10^{-3} mmol, 0.01 eq) of *chloro [1,3-bis(2',4',6'-diisopropylphenyl)imidazol-2-ylidene]gold(I)* (**1Cl**), 0.8 mg (8.8·10^{-3} mmol, 0.01 eq) of *silver tetrakis(3,5-bis(trifluoromethyl)phenyl)borate*, 4.4·10^{-2} mmol (0.05 eq) of *tetramethylethylene* and 550 uL (6.85 mmol, 7.78 eq) of deuterated *chloroform*.

5.5 Pseudo-catalysis experiment for pre-activated catalysts:

1Cl, 1OTf, 1OTs. A 4 mL vial was charged with 8.8·10^{-3} mmol (0.01 eq) of the appropriate NHC-gold(I) catalyst **(1X)**. Subsequently, 400 uL (5.00 mmol, 5.68 eq) of deuterated *chloroform*, 142 uL (3.52 mmol, 4.00 eq) of *methanol*, and 105 uL (0.88 mmol, 1.00 eq) of *tetramethylethylene* where added to the vessel which was placed under stirring till a homogeneous solution was formed. Finally, the solution was transferred to a 5 mm NMR tube for analysis.

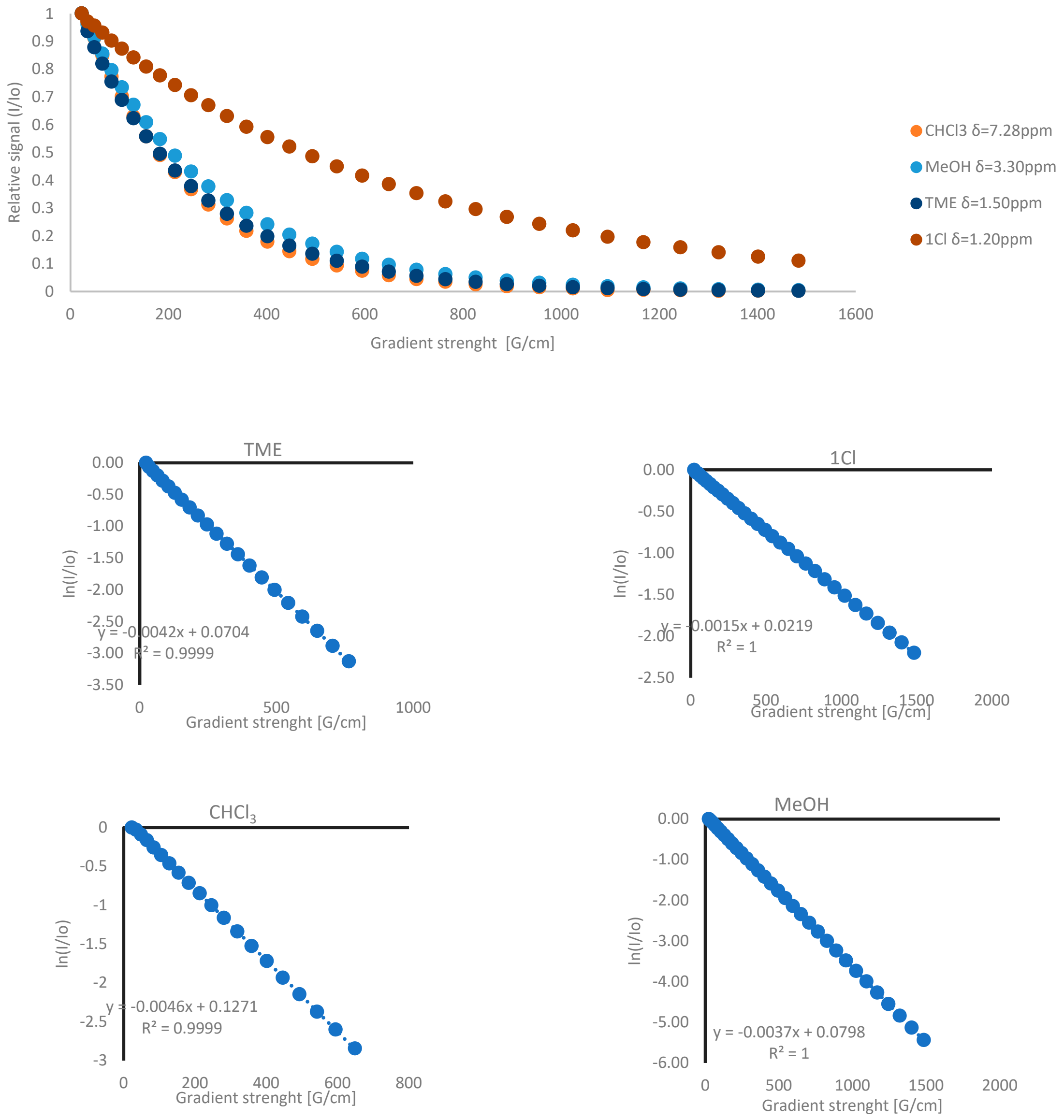

Figure S11 – Signal decay of the relevant chemical species, as observed during the ^{1}H-DOSY NMR experiment. The sample contained 5.46 mg (8.8 · 10^{-3} mmol, 0.01 eq) of *chloro [1,3-bis(2',4',6'-diisopropylphenyl)imidazol-2-ylidene]gold(I)* (**1Cl**), 400 uL (5.00 mmol, 5.68 eq) of deuterated *chloroform,* 142 uL (3.52 mmol, 4.00 eq) of *methanol*, and 105 uL (0.88 mmol, 1.00 eq) of *tetramethylethylene*.

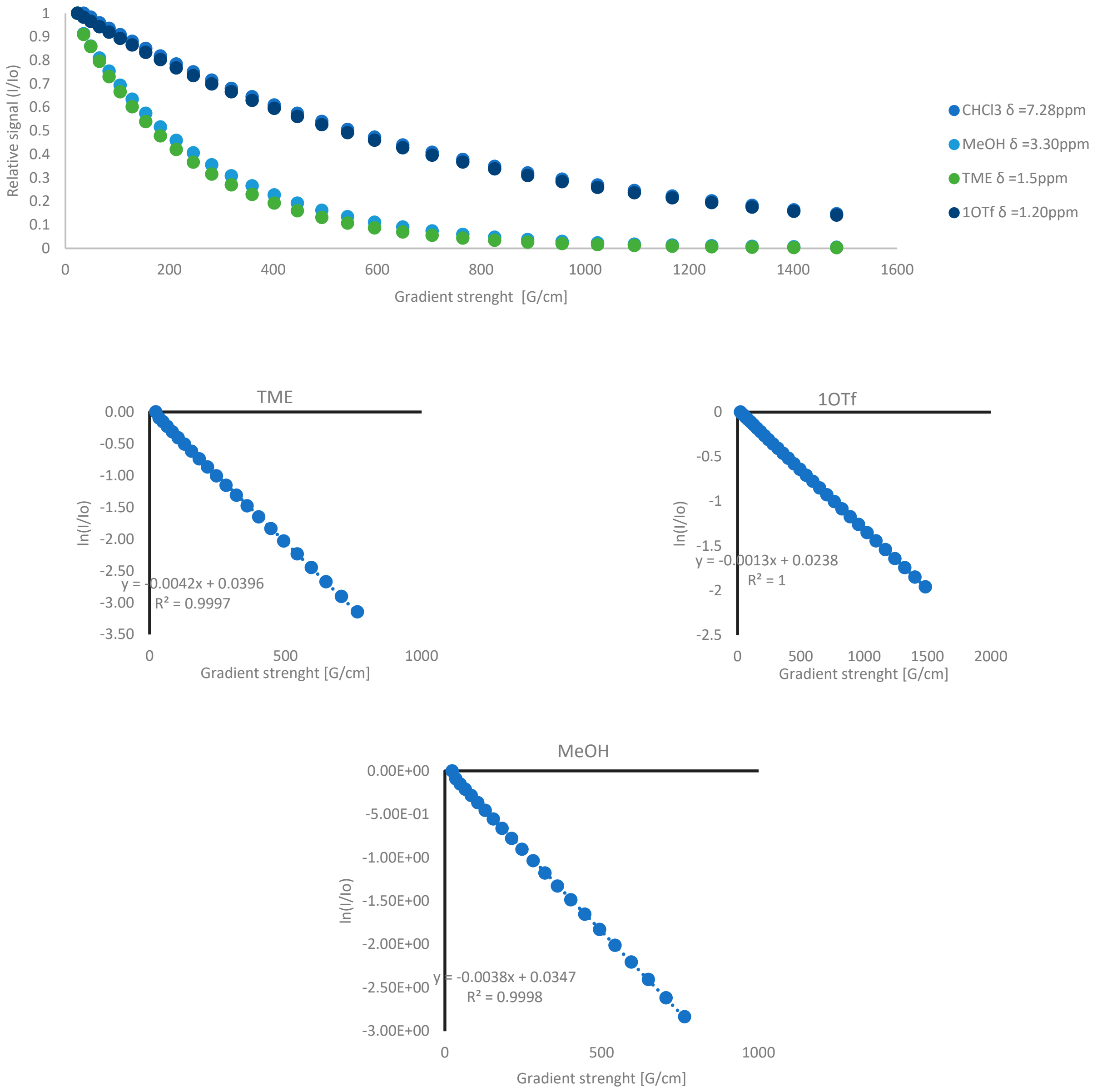

Figure S12 – Signal decay of the relevant chemical species, as observed during the ^{1}H-DOSY NMR experiment. The sample contained 6.46 mg (8.8 · 10^{-3} mmol, 0.01 eq) of *trifluoromethanesulfonate [1,3-bis(2',4',6'-diisopropylphenyl)imidazol-2-ylidene]gold(I)* (**1OTf**), 400 uL (5.00 mmol, 5.68 eq) of deuterated *chloroform,* 142 uL (3.52 mmol, 4.00 eq) of *methanol*, and 105 uL (0.88 mmol, 1.00 eq) of *tetramethylethylene*. Deuterated *chloroform*'s signal is covered by **1OTf** signal.

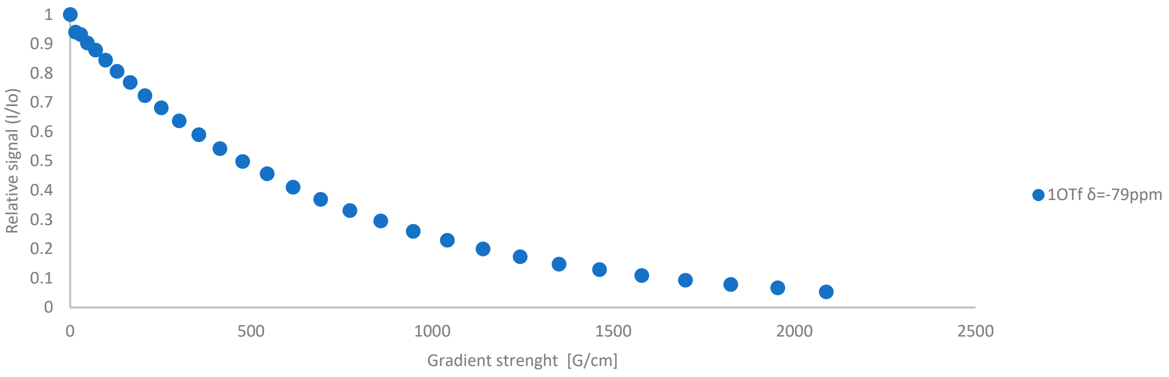

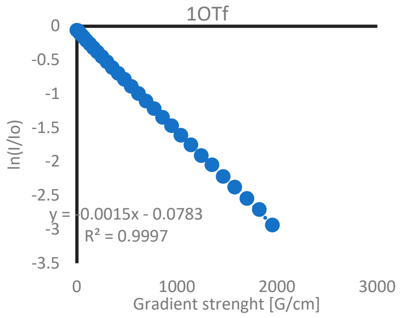

Figure S13 – Signal decay of the relevant chemical species, as observed during the ^{19}F-DOSY NMR experiment. The sample contained 6.46 mg (8.8 · 10^{-3} mmol, 0.01 eq) of *trifluoromethanesulfonate [1,3-bis(2',4',6'-diisopropylphenyl)imidazol-2-ylidene]gold(I)* (**1OTf**), 400 uL (5.00 mmol, 5.68 eq) of deuterated *chloroform,* 142 uL (3.52 mmol, 4.00 eq) of *methanol*, and 105 uL (0.88 mmol, 1.00 eq) of *tetramethylethylene.*

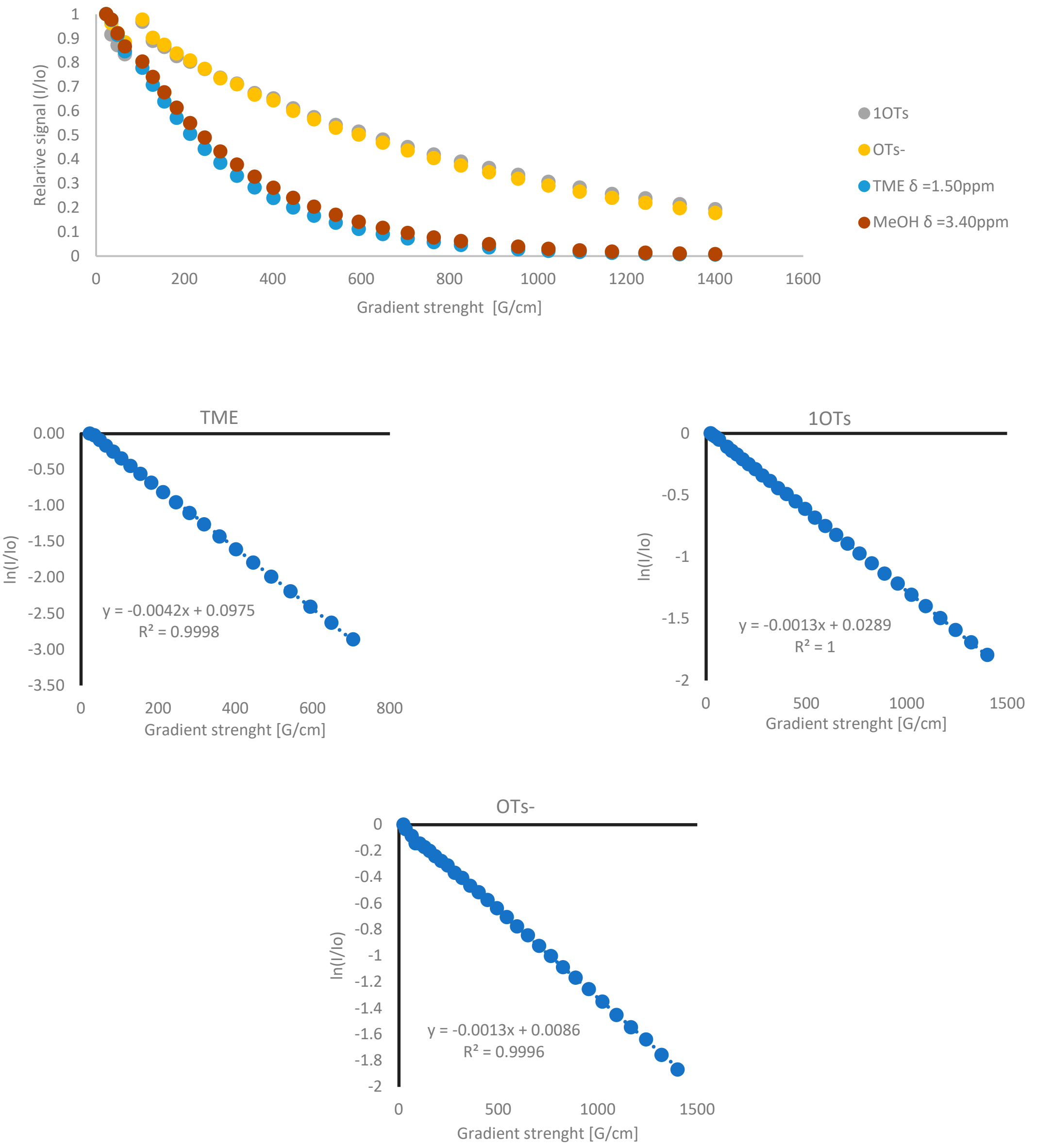

Figure S10 – Signal decay of the relevant chemical species, as observed during the ^{1}H-DOSY NMR experiment. The sample contained 6.46 mg (8.8 · 10^{-3} mmol, 0.01 eq) of *p-toluenesulfonate [1,3-bis(2',4',6'-diisopropylphenyl)imidazol-2-ylidene]gold(I)* (**1OTs**), 400 uL (5.00 mmol, 5.68 eq) of deuterated *chloroform,* 142 uL (3.52 mmol, 4.00 eq) of *methanol,* and 105 uL (0.88 mmol, 1.00 eq) of *tetramethylethylene.*

5.6 Pseudo-catalysis experiment involving *in situ* catalyst activation:

1(TME)BF$_4$, 1(TME)BARF. A 4 mL vial was charged under argon with 8.8·10^{-3} mmol (0.01 eq) of the appropriate silver salt, then 5.46 mg (8.8 · 10^{-3} mmol, 0.01 eq) of *chloro[1,3-bis(2',4',6'-diisopropylphenyl)imidazol-2-ylidene]gold(I)* (**1Cl**) were added. Subsequently, 400 uL (5.00 mmol, 5.68 eq) of deuterated *chloroform,* 142 uL (3.52 mmol, 4.00 eq) of *methanol*, and 105 uL (0.88 mmol, 1.00 eq) of *2,3-dimethyl-2-butene* where added to the vessel. The reacting mixture was left stirring for 1 h while sealed and shielded from light at RT. After decanting the solid residue, the solution was transferred to a 5 mm NMR tube for analysis.

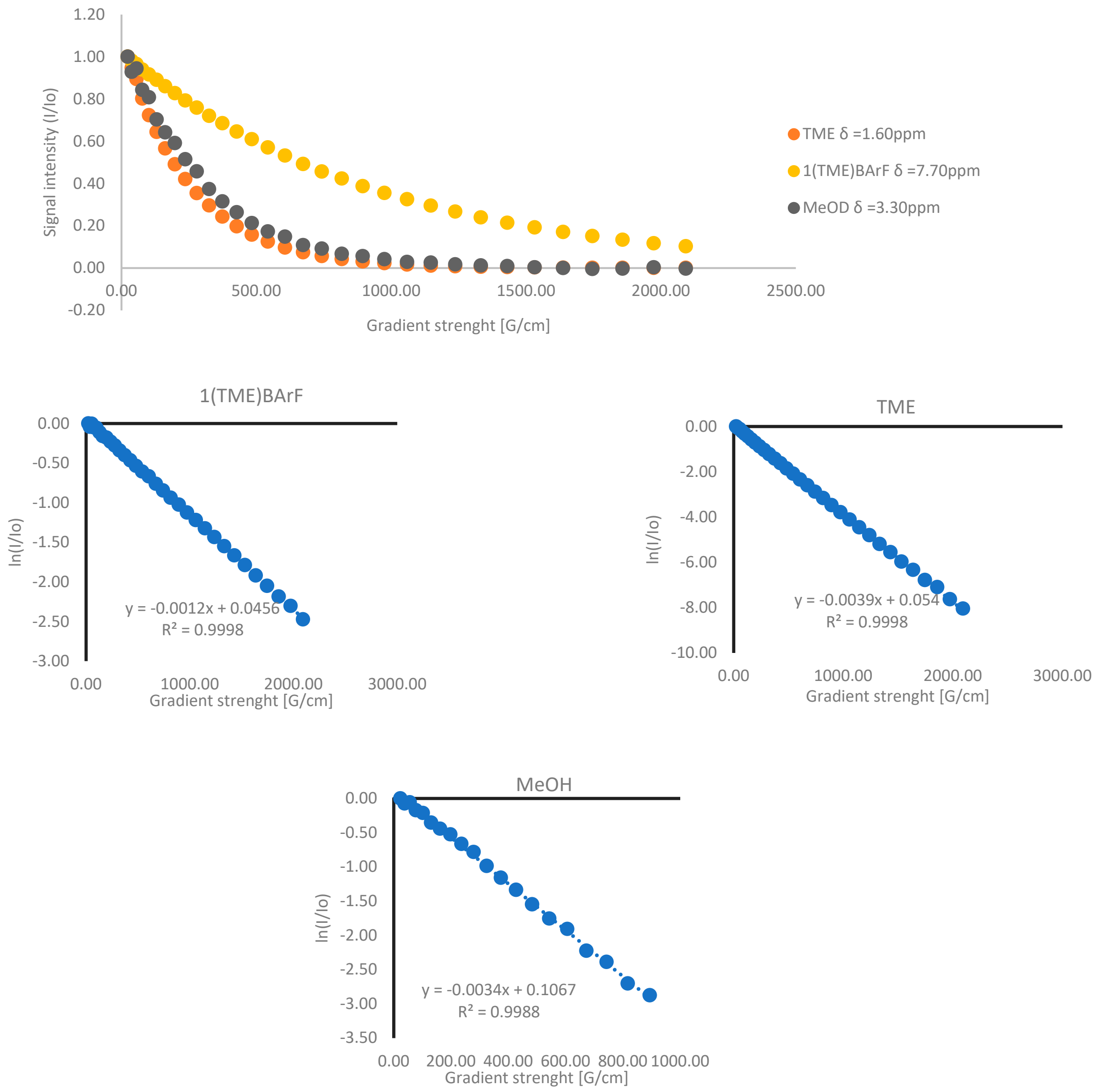

Figure S14 – Signal decay of the relevant chemical species, as observed during the ^{1}H-DOSY NMR experiment. The sample contained 5.46 mg (8.8 · 10^{-3} mmol, 0.01 eq) of *chloro[1,3-bis(2',4',6'-diisopropylphenyl)imidazol-2-ylidene]gold(I)* (**1Cl**), 0.8 mg (8.8·10^{-3} mmol, 0.01 eq) of *silver tetrakis(3,5-bis(trifluoromethyl)phenyl)borate*, 400 uL (5.00 mmol, 5.68 eq) of deuterated *chloroform,* 142 uL (3.52 mmol, 4.00 eq) of *methanol*, and 105 uL (0.88 mmol, 1.00 eq) of *tetramethylethylene.*

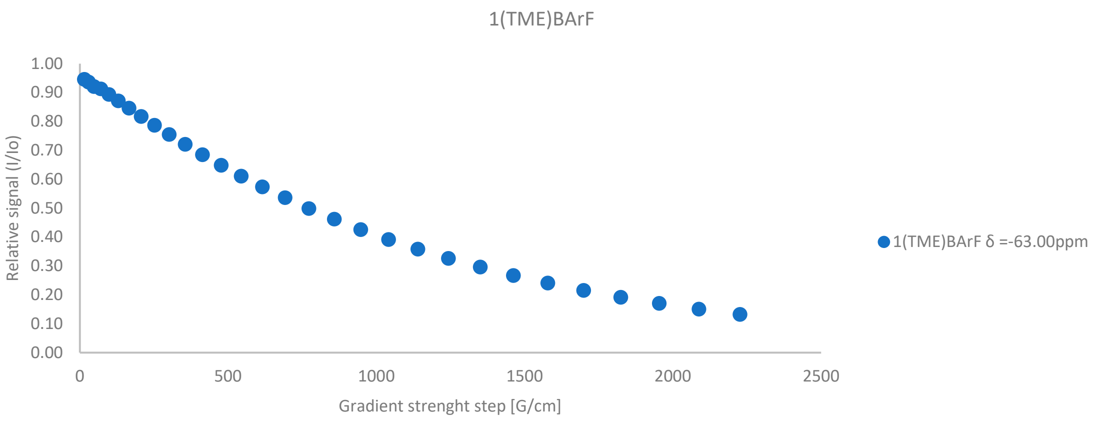

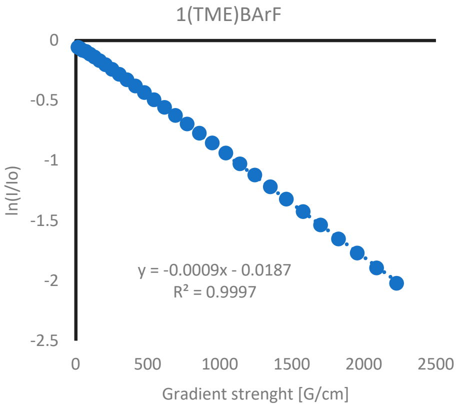

Figure S15 – Signal decay of the relevant chemical species, as observed during the ^{19}F-DOSY NMR experiment. The sample contained 5.46 mg (8.8 · 10^{-3} mmol, 0.01 eq) of *chloro[1,3-bis(2',4',6'-diisopropylphenyl)imidazol-2-ylidene]gold(I)* (**1Cl**), 0.8 mg (8.8·10^{-3} mmol, 0.01 eq) of *silver tetrakis(3,5-bis(trifluoromethyl)phenyl)borate*, 400 uL (5.00 mmol, 5.68 eq) of deuterated *chloroform,* 142 uL (3.52 mmol, 4.00 eq) of *methanol*, and 105 uL (0.88 mmol, 1.00 eq) of *tetramethylethylene*.

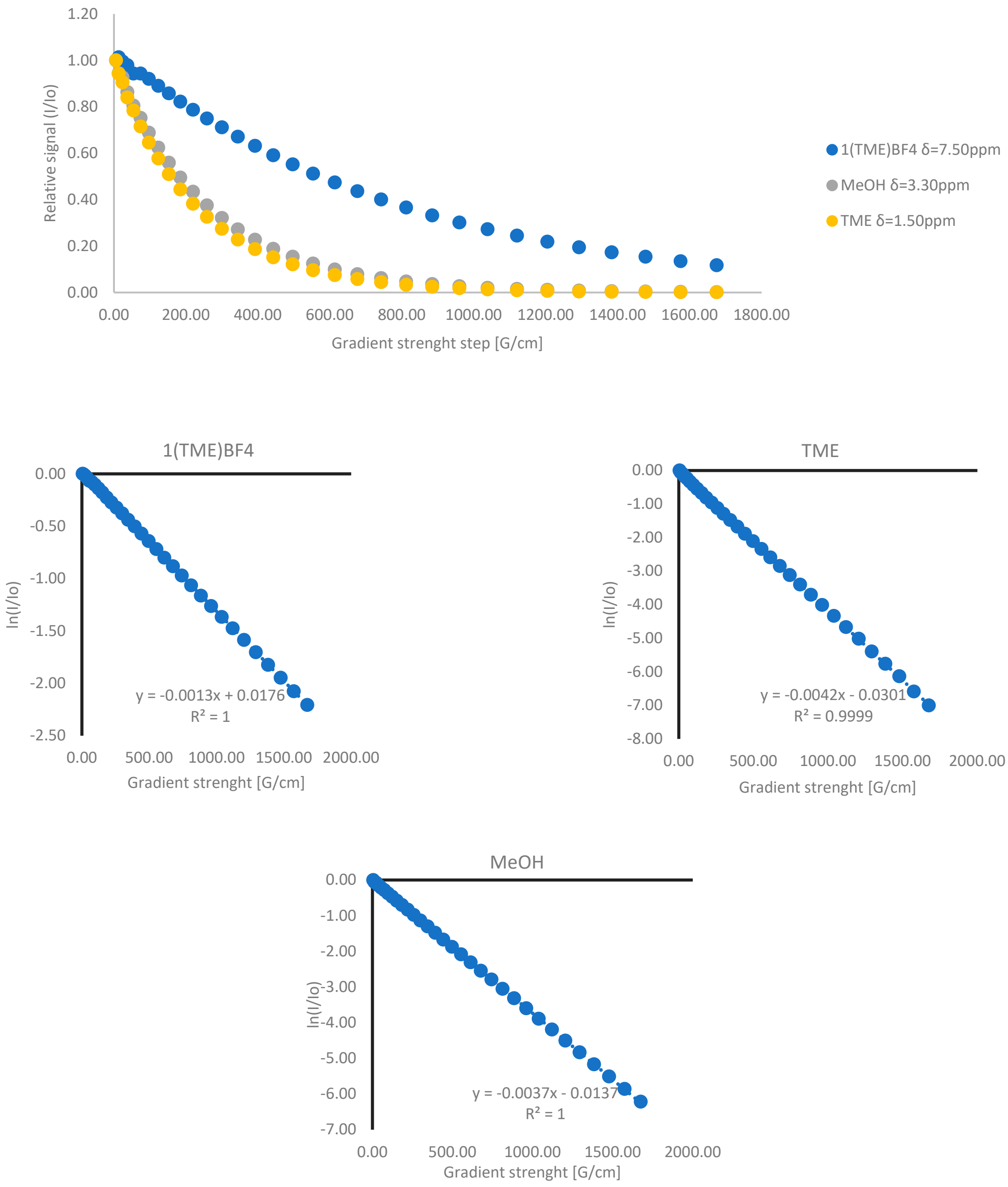

Figure S16 – Signal decay of the relevant chemical species, as observed during the ^{1}H-DOSY NMR experiment. The sample contained 5.46 mg ($8.8 \cdot 10^{-3}$ mmol, 0.01 eq) of *chloro[1,3-bis(2',4',6'-diisopropylphenyl)imidazol-2-ylidene]gold(I)* (**1Cl**), 0.3 mg ($8.8 \cdot 10^{-3}$ mmol, 0.01 eq) of *silver tetrafluoroborate*, 400 uL (5.00 mmol, 5.68 eq) of deuterated *chloroform,* 142 uL (3.52 mmol, 4.00 eq) of *methanol*, and 105 uL (0.88 mmol, 1.00 eq) of *tetramethylethylene.*

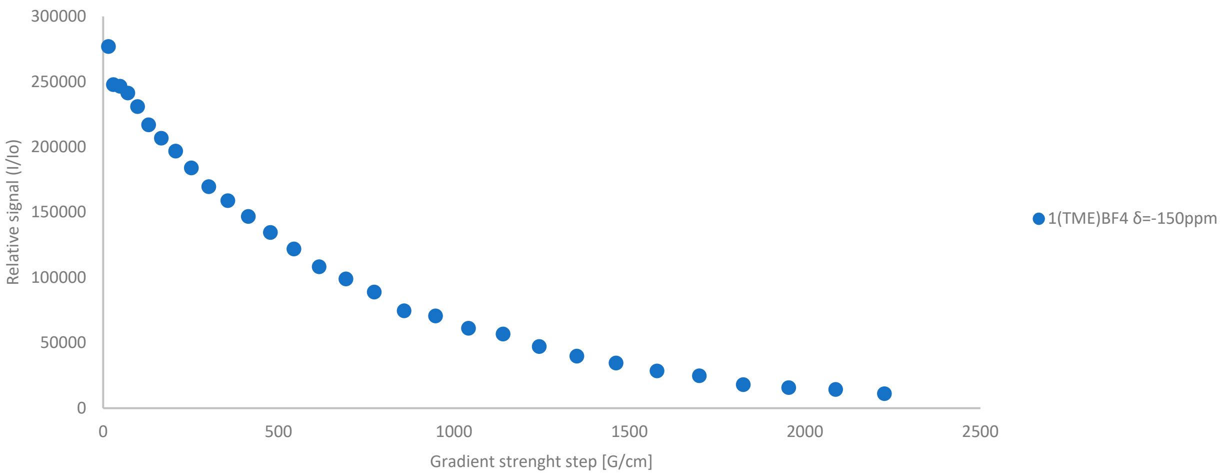

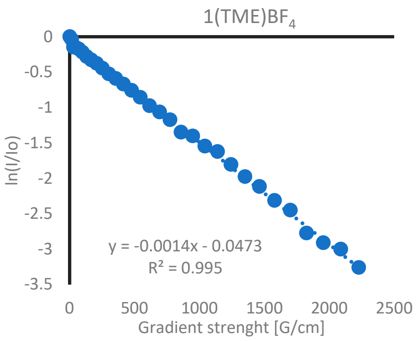

Figure S17 – Signal decay of the relevant chemical species, as observed during the ^{19}F-DOSY NMR experiment. The sample contained 5.46 mg ($8.8 \cdot 10^{-3}$ mmol, 0.01 eq) of *chloro[1,3-bis(2',4',6'-diisopropylphenyl)imidazol-2-ylidene]gold(I)* (**1Cl**), 0.3 mg ($8.8 \cdot 10^{-3}$ mmol, 0.01 eq) of *silver tetrakis(3,5-bis(trifluoromethyl)phenyl)borate*, 400 uL (5.00 mmol, 5.68 eq) of deuterated *chloroform,* 142 uL (3.52 mmol, 4.00 eq) of *methanol*, and 105 uL (0.88 mmol, 1.00 eq) of *tetramethylethylene*.

5.7 Pseudo-catalysis experiment involving different solvents:

1OTf. A 4 mL vial was charged with 6.46 mg (8.8 · 10-3 mmol, 0.01 eq) of *trifluoromethanesulfonate [1,3-bis(2',4',6'-diisopropylphenyl)imidazol-2-ylidene]gold(I)* (**1OTf**). Subsequently, 400 uL (5.00 mmol, 5.68 eq) of the selected solvent, 142 uL (3.52 mmol, 4.00 eq) of deuterated *methanol*, and 105 uL (0.88 mmol, 1.00 eq) of *tetramethylethylene* where added to the vessel which was placed under stirring till a homogeneous solution was formed. Finally, the solution was transferred to a 5 mm NMR tube for analysis.

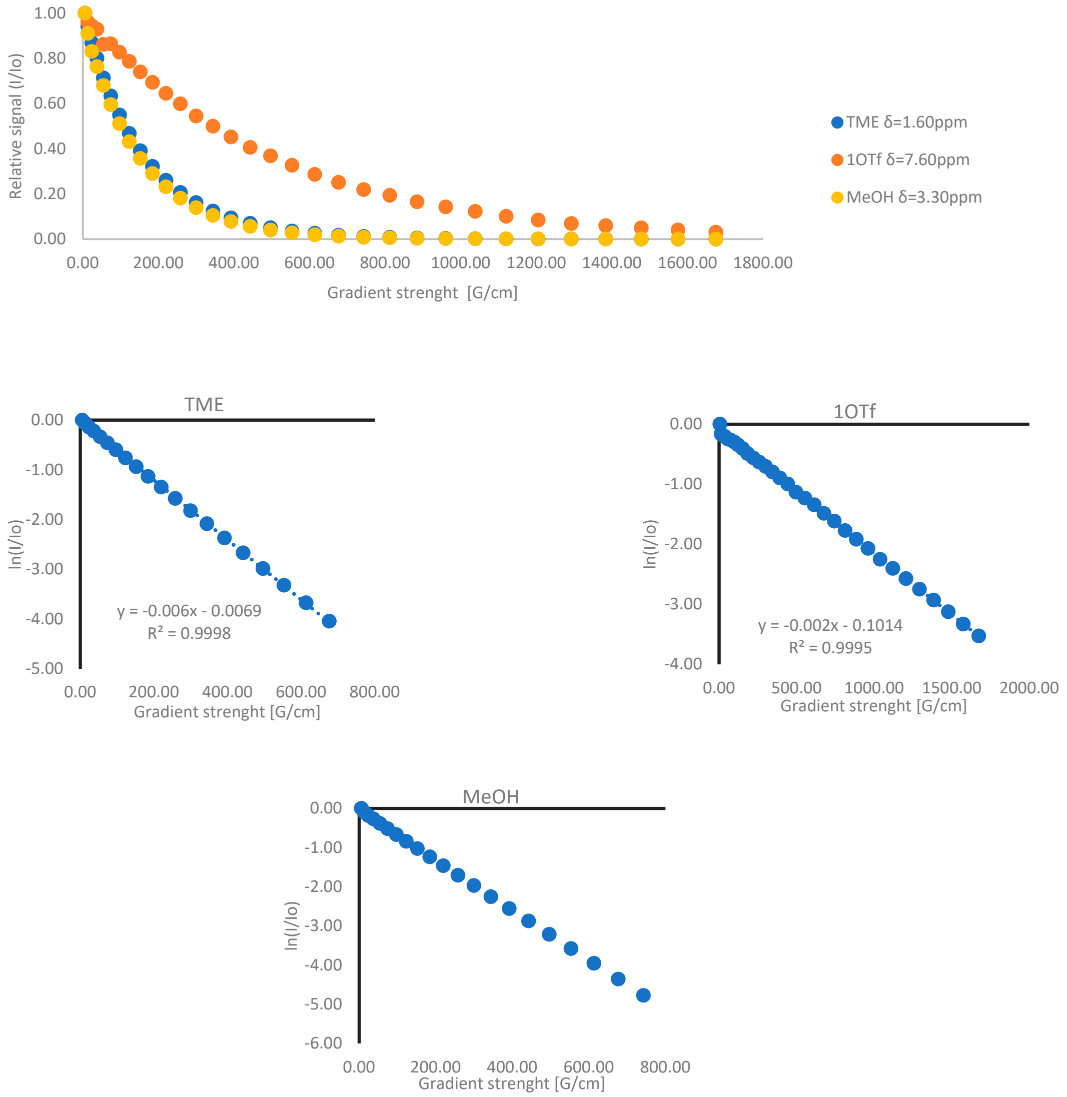

Figure S18 – Signal decay of the relevant chemical species, as observed during the ^{1}H-DOSY NMR experiment. The sample contained 6.46 mg (8.8 · 10-3 mmol, 0.01 eq) of *trifluoromethansulfonate [1,3-bis(2',4',6'-diisopropylphenyl)imidazol-2-ylidene]gold(I)* (**1OTf**), 400 uL (5.00 mmol, 5.68 eq) of deuterated *acetone,* 142 uL (3.52 mmol, 4.00 eq) of *methanol*, and 105 uL (0.88 mmol, 1.00 eq) of *tetramethylethylene.*

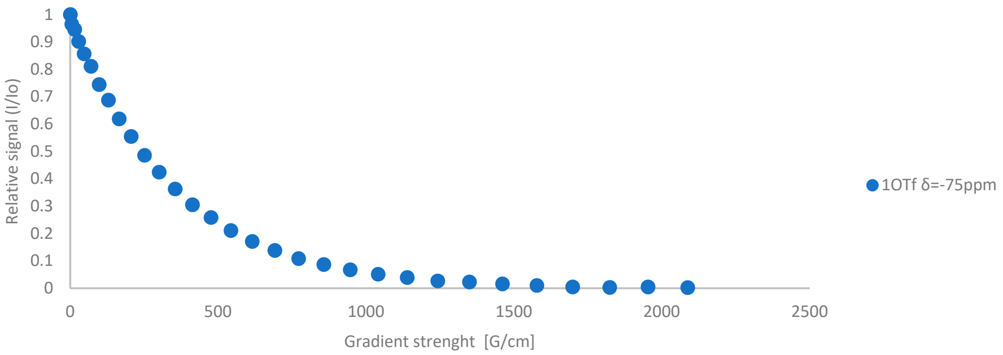

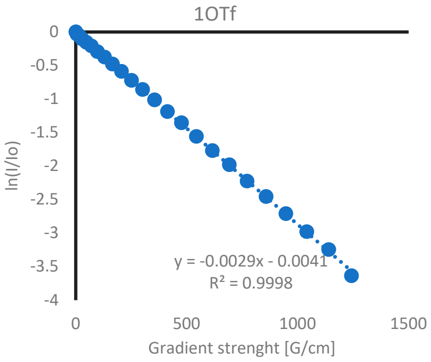

Figure S19 – Signal decay of the relevant chemical species, as observed during the ^{19}F-DOSY NMR experiment. The sample contained 6.46 mg (8.8 · 10^{-3} mmol, 0.01 eq) of *trifluoromethansulfonate [1,3-bis(2',4',6'-diisopropylphenyl)imidazol-2-ylidene]gold(I)* (**1OTf**), 400 uL (5.00 mmol, 5.68 eq) of deuterated *acetone,* 142 uL (3.52 mmol, 4.00 eq) of *methanol*, and 105 uL (0.88 mmol, 1.00 eq) of *tetramethylethylene*.

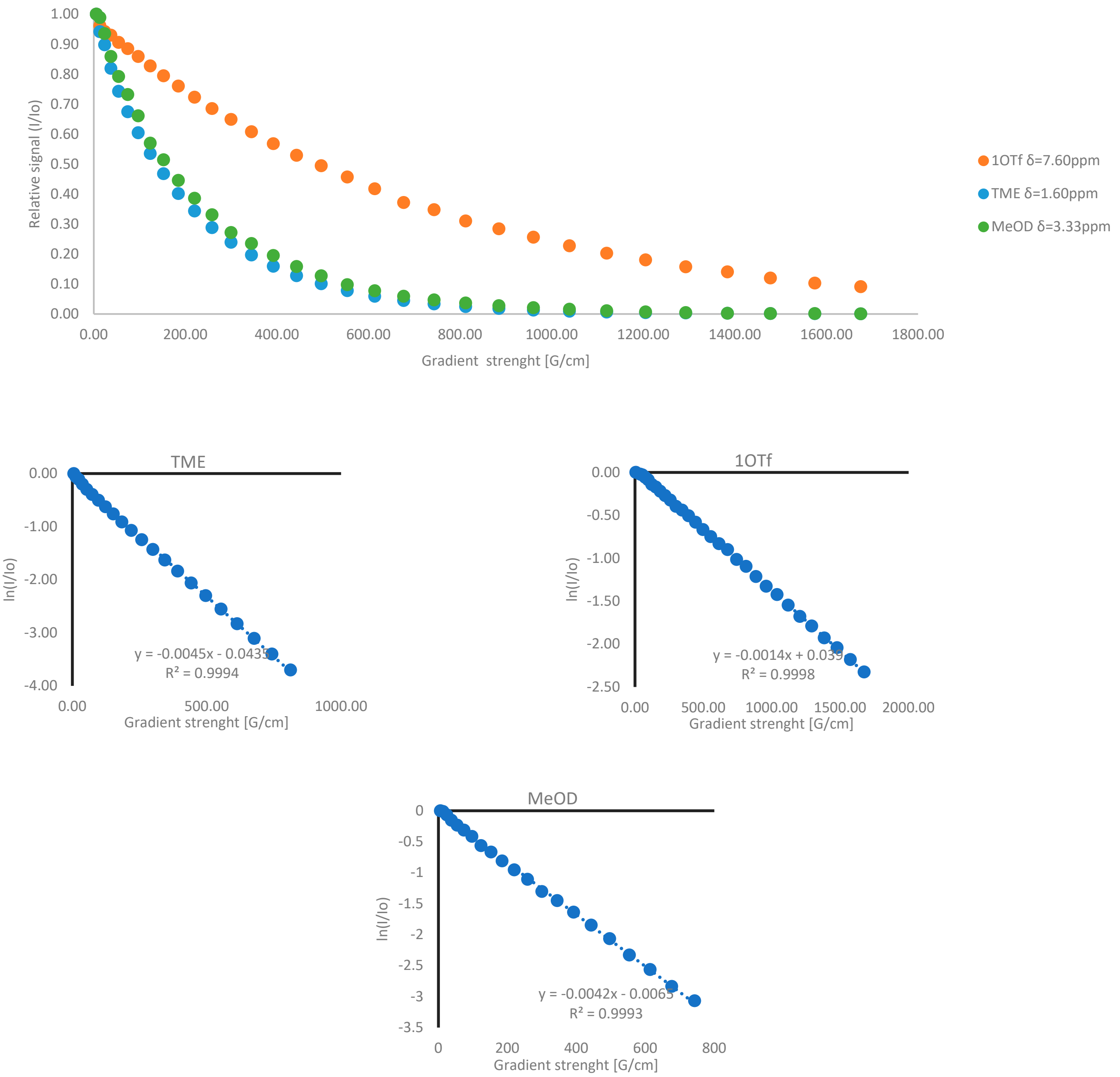

Figure S20 – Signal decay of the relevant chemical species, as observed during the ^{1}H-DOSY NMR experiment. The sample contained 6.46 mg (8.8 · 10^{-3} mmol, 0.01 eq) of *trifluoromethansulfonate [1,3-bis(2',4',6'-diisopropylphenyl)imidazol-2-ylidene]gold(I)* (**1OTf**), 542 uL (13.41 mmol, 15.00 eq) of deuterated *methanol*, and 105 uL (0.88 mmol, 1.00 eq) of *tetramethylethylene.*

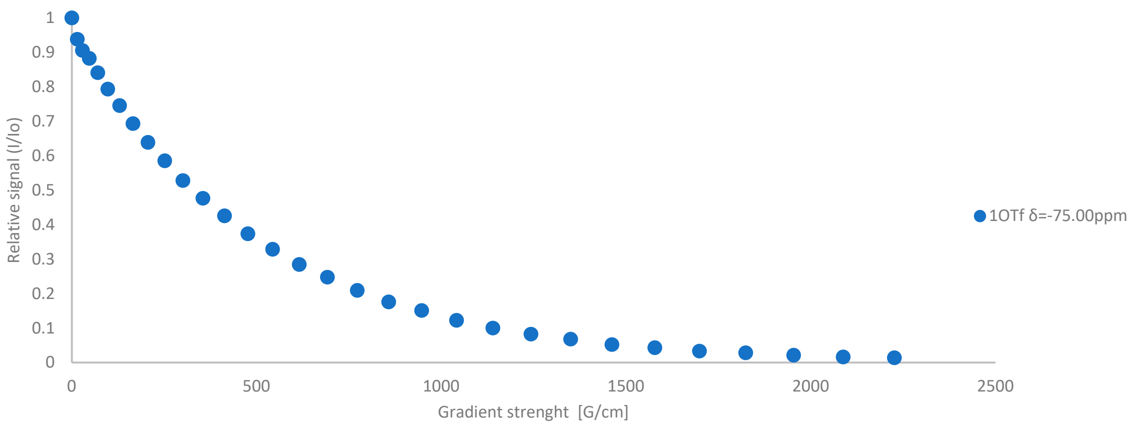

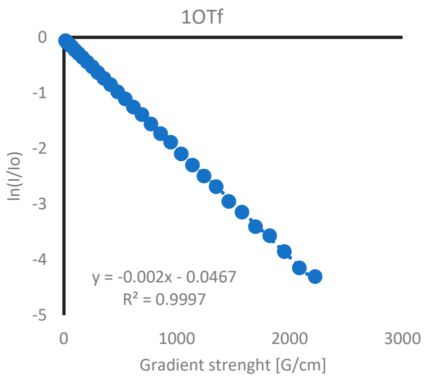

Figure S21 – Signal decay of the relevant chemical species, as observed during the ^{19}F-DOSY NMR experiment. The sample contained 6.46 mg (8.8 · 10^{-3} mmol, 0.01 eq) of *trifluoromethansulfonate [1,3-bis(2',4',6'-diisopropylphenyl)imidazol-2-ylidene]gold(I)* (**1OTf**), 542 uL (13.41 mmol, 15.00 eq) of deuterated *methanol*, and 105 uL (0.88 mmol, 1.00 eq) of *tetramethylethylene*.

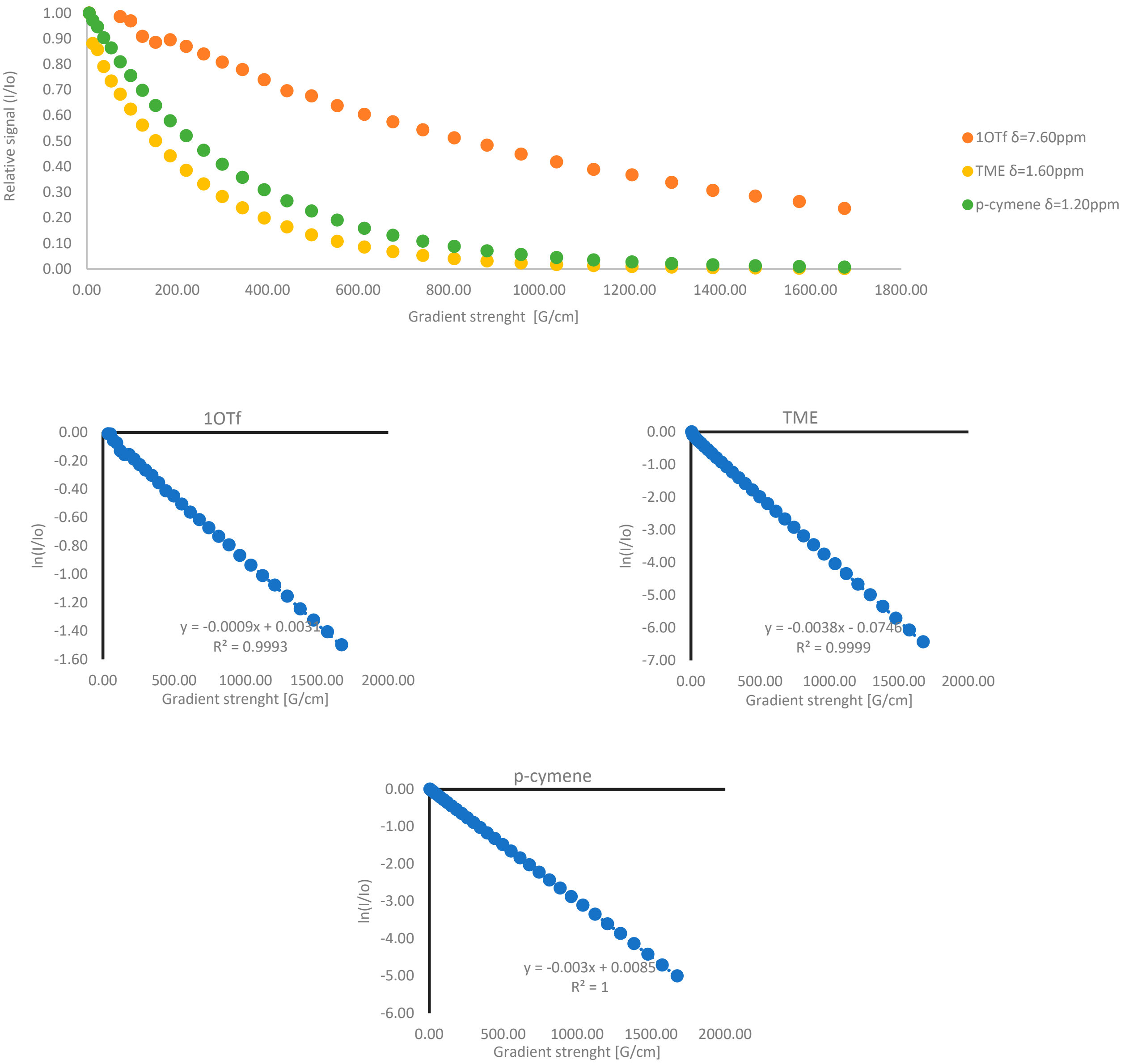

Figure S22 – Signal decay of the relevant chemical species, as observed during the ^{1}H-DOSY NMR experiment. The sample contained 6.46 mg ($8.8 \cdot 10^{-3}$ mmol, 0.01 eq) of *trifluoromethansulfonate [1,3-bis(2',4',6'-diisopropylphenyl)imidazol-2-ylidene]gold(I)* (**1OTf**), 400 uL (2.59 mmol, 2.95 eq) of *p-cymene,* 142 uL (3.52 mmol, 4.00 eq) of deuterated *methanol*, and 105 uL (0.88 mmol, 1.00 eq) of *tetramethylethylene.*

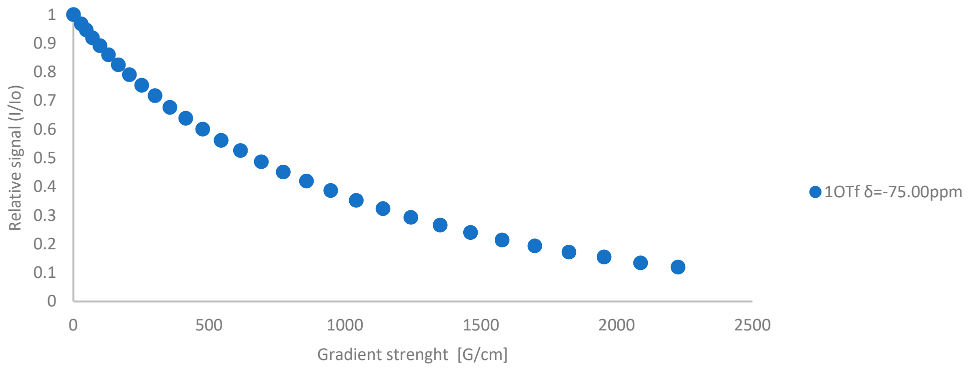

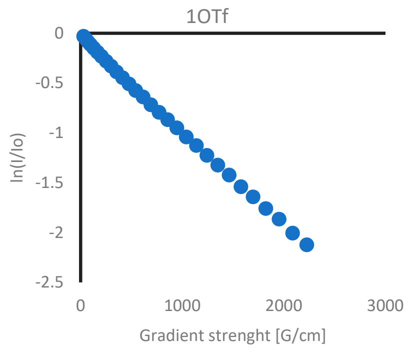

Figure S23 – Signal decay of the relevant chemical species, as observed during the ^{19}F-DOSY NMR experiment. The sample contained 6.46 mg (8.8 · 10^{-3} mmol, 0.01 eq) of *trifluoromethansulfonate [1,3-bis(2',4',6'-diisopropylphenyl)imidazol-2-ylidene]gold(I)* (**1OTf**), 400 uL (2.59 mmol, 2.95 eq) of *p-cymene,* 142 uL (3.52 mmol, 4.00 eq) of deuterated *methanol*, and 105 uL (0.88 mmol, 1.00 eq) of *tetramethylethylene*.

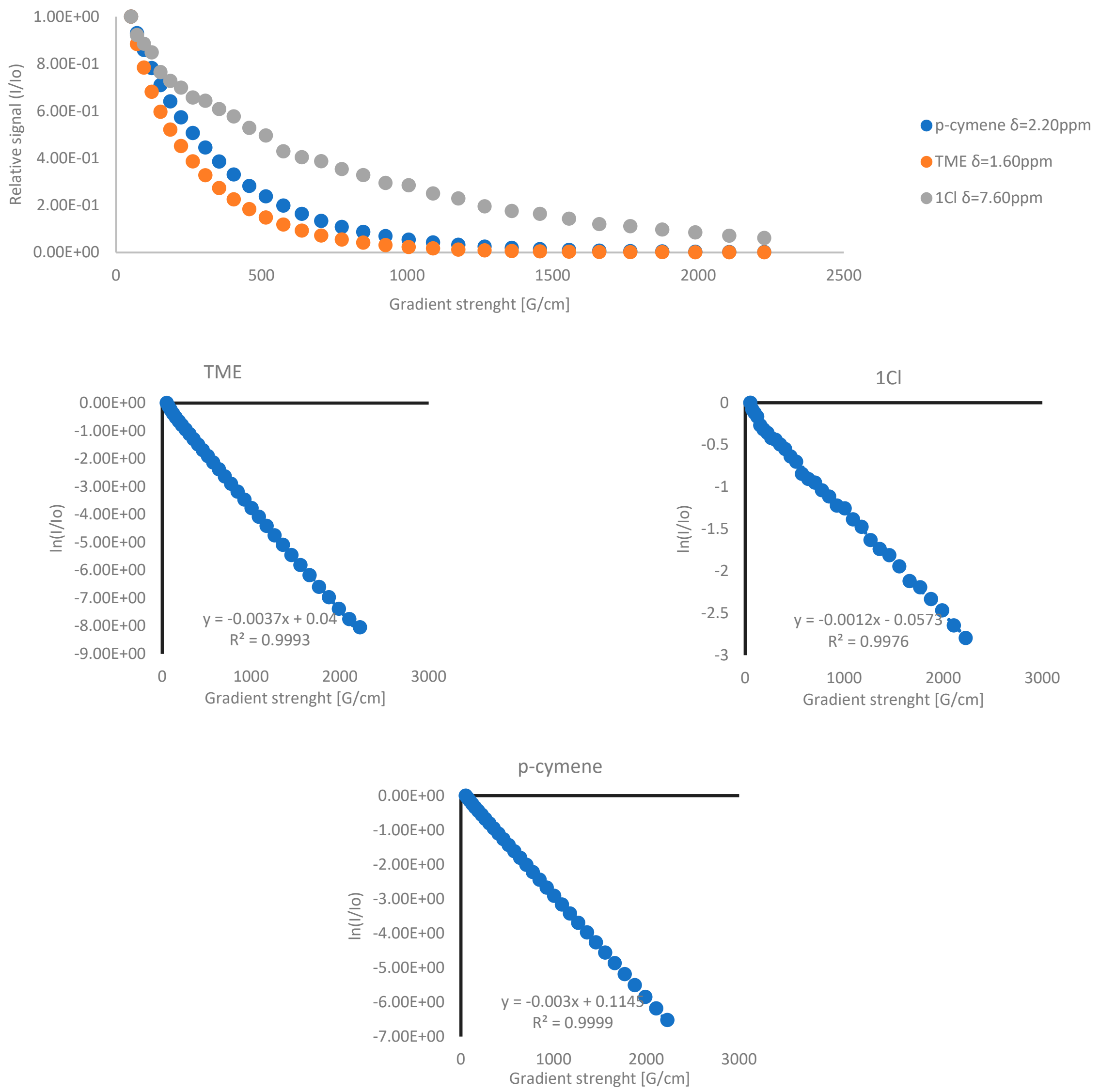

Figure S24 – Signal decay of the relevant chemical species, as observed during the ^{1}H-DOSY NMR experiment. The sample contained 5.46 mg ($8.8 \cdot 10^{-3}$ mmol, 0.01 eq) of *chloro [1,3-bis(2',4',6'-diisopropylphenyl)imidazol-2-ylidene]gold(I)* (**1Cl**), 400 uL (2.59 mmol, 2.95 eq) of *p-cymene,* 142 uL (3.52 mmol, 4.00 eq) of deuterated *methanol*, and 105 uL (0.88 mmol, 1.00 eq) of *tetramethylethylene.*

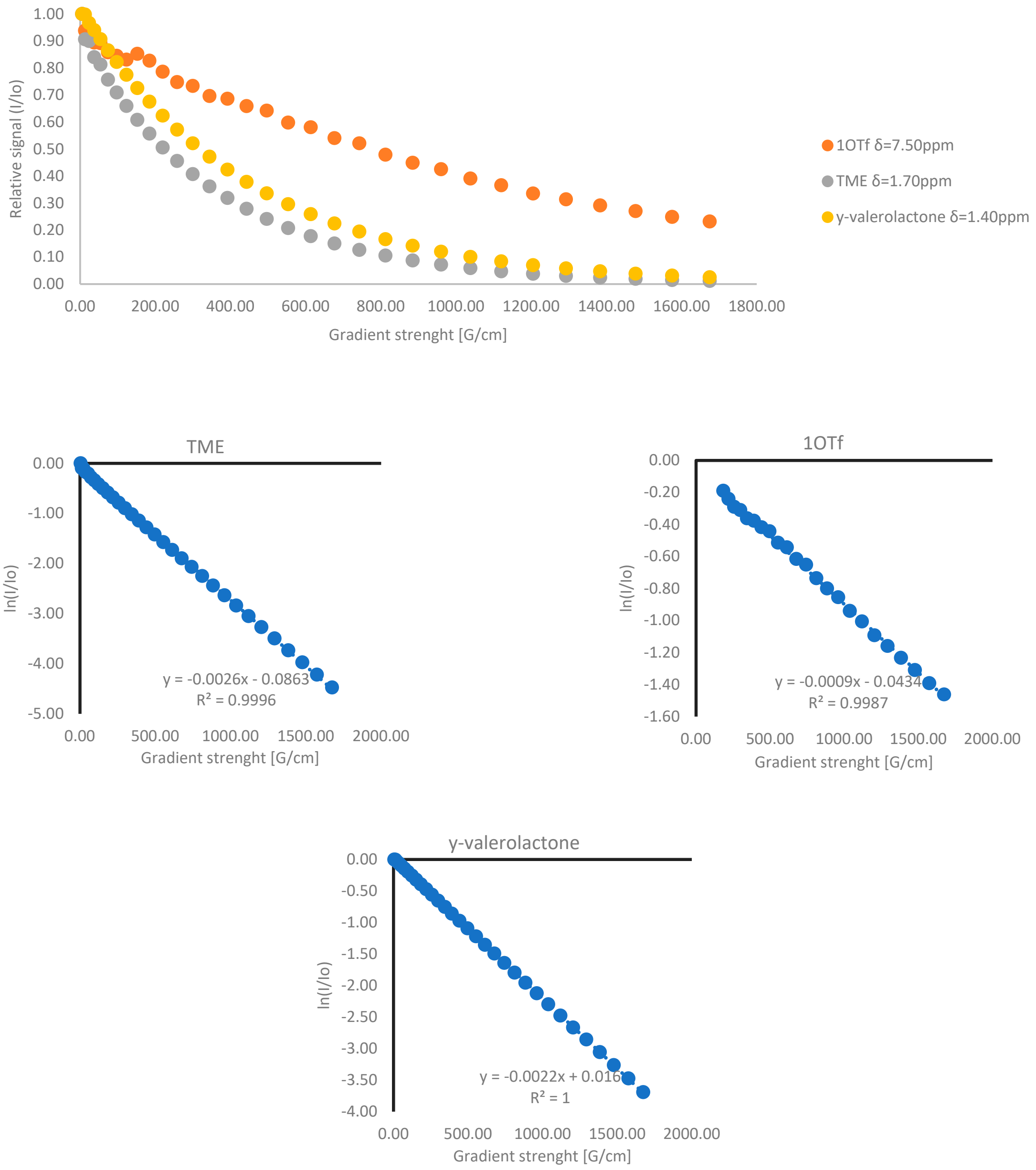

Figure S25 – Signal decay of the relevant chemical species, as observed during the ^{1}H-DOSY NMR experiment. The sample contained 6.46 mg ($8.8 \cdot 10^{-3}$ mmol, 0.01 eq) of *trifluoromethansulfonate [1,3-bis(2',4',6'-diisopropylphenyl)imidazol-2-ylidene]gold(I)* (**1OTf**), 400 uL (4.2 mmol, 4.77 eq) of *γ-valerolactone,* 142 uL (3.52 mmol, 4.00 eq) of deuterated *methanol*, and 105 uL (0.88 mmol, 1.00 eq) of *tetramethylethylene.*

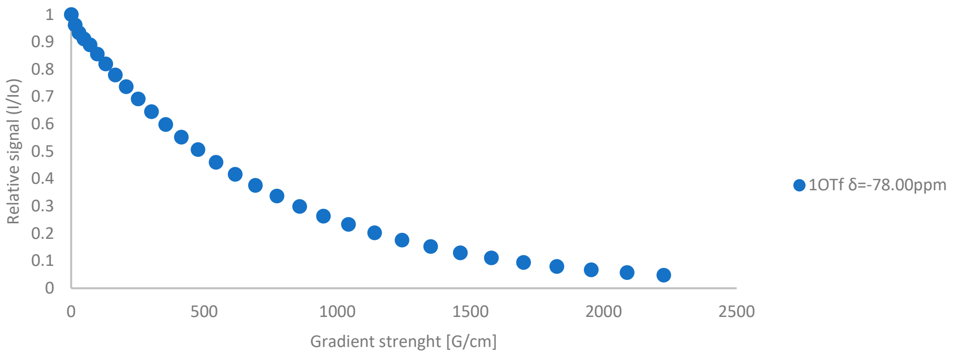

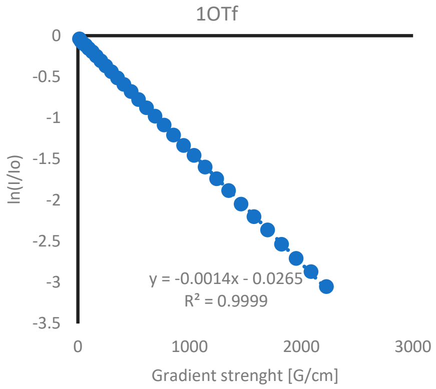

Figure S26 – Signal decay of the relevant chemical species, as observed during the ^{19}F-DOSY NMR experiment. The sample contained 6.46 mg (8.8 · 10^{-3} mmol, 0.01 eq) of *trifluoromethansulfonate [1,3-bis(2',4',6'-diisopropylphenyl)imidazol-2-ylidene]gold(I)* (**1OTf**), 400 uL (4.2 mmol, 4.77 eq) of *γ-valerolactone,* 142 uL (3.52 mmol, 4.00 eq) of deuterated *methanol*, and 105 uL (0.88 mmol, 1.00 eq) of *tetramethylethylene*.

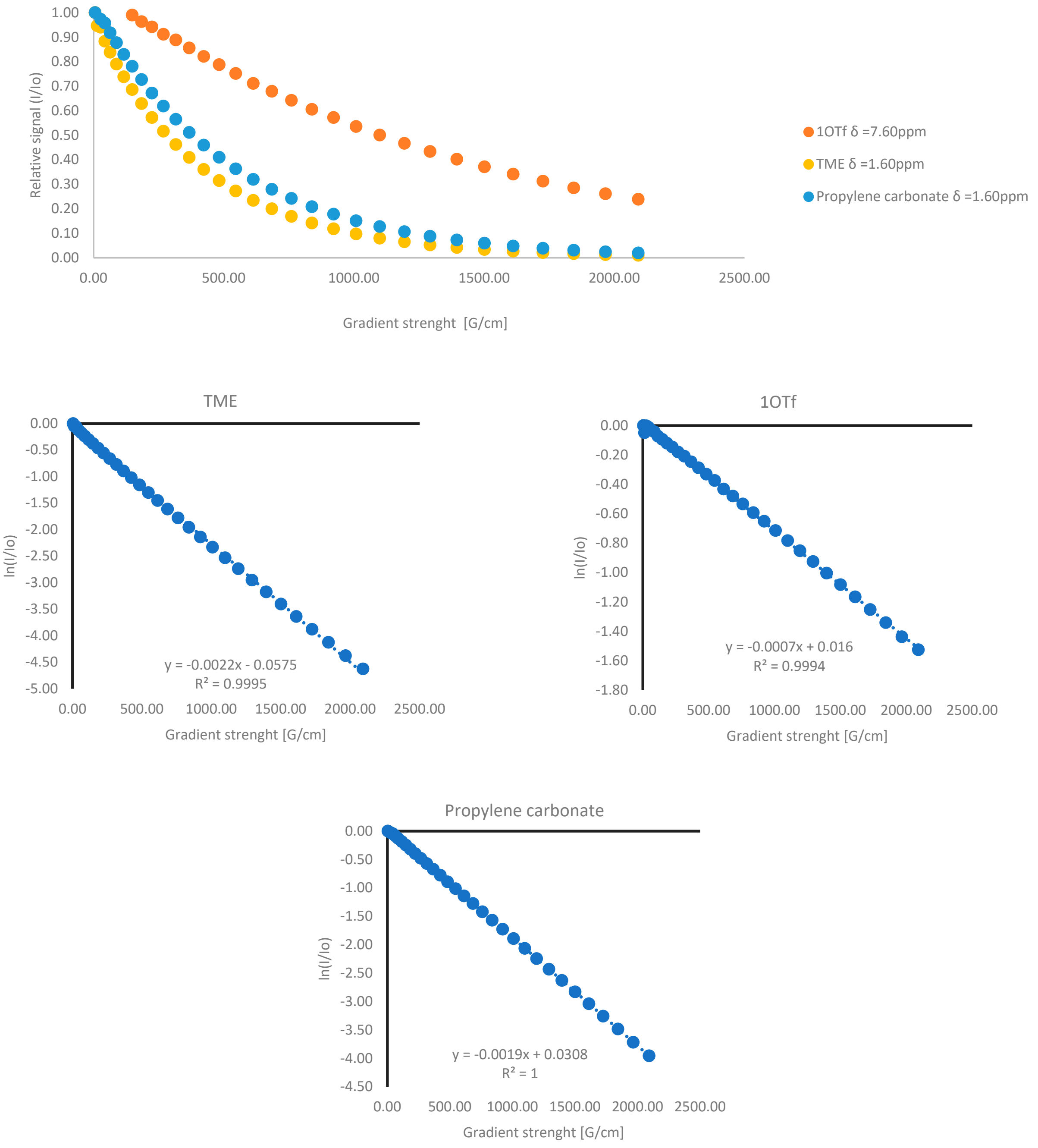

Figure S27 – Signal decay of the relevant chemical species, as observed during the ^{1}H-DOSY NMR experiment. The sample contained 6.46 mg (8.8 · 10^{-3} mmol, 0.01 eq) of *trifluoromethansulfonate [1,3-bis(2',4',6'-diisopropylphenyl)imidazol-2-ylidene]gold(I)* (**1OTf**), 400 uL (4.7 mmol, 5.34 eq) of *propylene carbonate,* 142 uL (3.52 mmol, 4.00 eq) of deuterated *methanol*, and 105 uL (0.88 mmol, 1.00 eq) of *tetramethylethylene*.

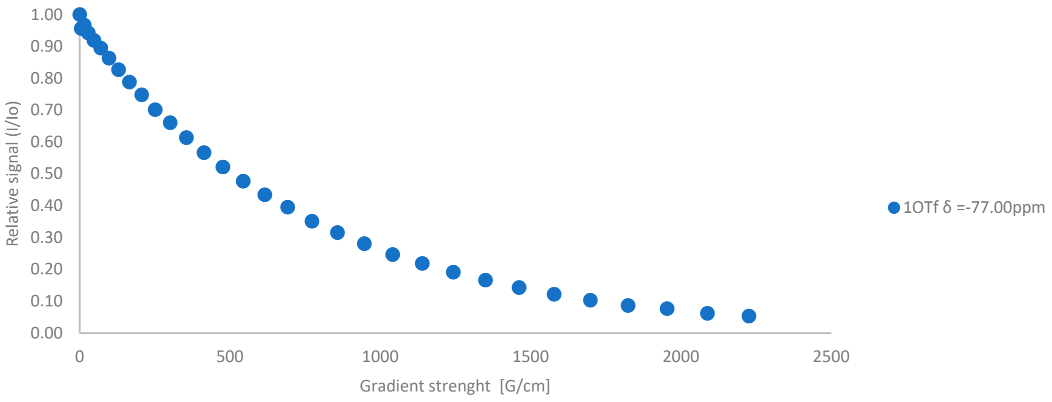

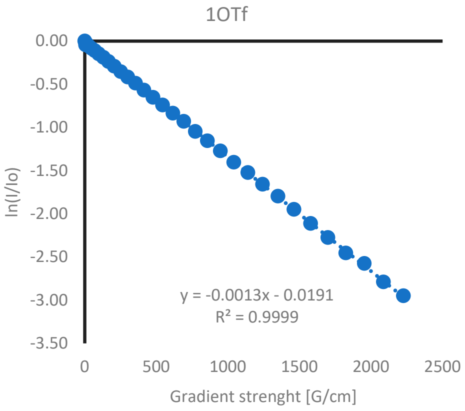

Figure S28 – Signal decay of the relevant chemical species, as observed during the ^{19}F-DOSY NMR experiment. The sample contained 6.46 mg (8.8 · 10^{-3} mmol, 0.01 eq) of *trifluoromethansulfonate [1,3-bis(2',4',6'-diisopropylphenyl)imidazol-2-ylidene]gold(I)* (**1OTf**), 400 uL (4.7 mmol, 5.34 eq) of *propylene carbonate,* 142 uL (3.52 mmol, 4.00 eq) of deuterated *methanol*, and 105 uL (0.88 mmol, 1.00 eq) of *tetramethylethylene.*

6 Data analysis

6.1 Calculated hydrodynamic volumes in pure chloroform

Table S2 – Summary of the hydrodynamic volumes (V_H) of the catalytic species (**1X**) obtained for each catalyst in pure chloroform ($CDCl_3$). The experiments were carried out following the disclosed procedure (Sections 5.2 and 5.4). The volumes were obtained following the disclosed procedure in Section 5.1

Compound	D_t ($CDCl_3$)	Error	m ($CDCl_3$)	D_t (X^-)	Error	m(X^-)	r(X^-)	V (X^-)	D_t (1^+)	Error	m(1^+)	r(1^+)	V (1^+)
[Code]	[m^2/s]	[%]	[u]	[m^2/s]	[%]	[u]	[A]	[A^3]	[m^2/s]	[%]	[u]	[A]	[A^3]
**1**OTf	1.87E-09	13.16	-4.18E-03	7.36E-10	1.42	-1.56E-03	5.1	659	6.78E-10	5.99	-1.45E-03	5.4	732
**1**OTs	2.07E-09	1.94	-4.08E-03	6.81E-10	4.83	-1.33E-03	5.7	780	6.29E-10	2.22	-1.30E-03	5.8	809
**1(TME)**BF_4	2.44E-09	2.45	-4.15E-03	6.60E-10	3.30	-1.30E-03	5.9	847	6.32E-10	3.55	-1.33E-03	5.8	805
**1(TME)**BARF	2.04E-09	2.14	-4.38E-03	5.58E-10	0.96	-1.10E-03	7.1	1487	5.85E-10	0.75	-1.19E-03	6.6	1204

Table S3 – Summary of the hydrodynamic volumes (V_H) of the catalytic species (**1X**) obtained for each catalyst under pseudo-catalytic conditions. The experiments were carried out following the disclosed procedure (Sections 5.2, 5.5 and 5.6). The volumes were obtained following the disclosed procedure in Section 5.1

Compound	D_t (C_6H_{12})	Error	m ($CDCl_3$)	D_t (X^-)	Error	m(X^-)	r(X^-)	V (X^-)	D_t (1^+)	Error	m(1^+)	r(1^+)	V (1^+)
[Code]	[m^2/s]	[%]	[u]	[m^2/s]	[%]	[u]	[A]	[A^3]	[m^2/s]	[%]	[u]	[A]	[A^3]
**1**Cl	1.97E-09	1.1	-4.40E-03						6.99E-10	0.5	-1.49E-03	6.1	937
**1**OTf	2.02E-09	1.1	-4.34E-03	7.38E-10	0.3	-1.49E-03	6.0	900	6.40E-10	0.6	-1.43E-03	6.2	998
**1**OTs	1.99E-09	1.0	-4.41E-03	5.24E-10	3.2	-1.40E-03	6.4	1098	5.93E-10	1.5	-1.36E-03	6.6	1188
**1(TME)**BF_4	2.01E-09	2.0	-4.30E-03	7.65E-10	8.5	-1.62E-03	5.6	720	6.01E-10	1.3	-1.25E-03	6.9	1376
**1(TME)**BARF	2.26E-09	2.1	-3.99E-03	5.58E-10	0.6	-1.10E-03	7.3	1596	6.84E-10	1.7	-1.20E-03	6.7	1260

Table S4 – Summary of the hydrodynamic volumes (V_H) of the catalytic species (**1OTf**) obtained in different reaction media under pseudo-catalytic conditions. The experiments were carried out following the disclosed procedure (Sections 5.2 and 5.7). The volumes were obtained following the disclosed procedure in Section 5.1

Compound	D_t (C_6H_{12})	Error	m ($CDCl_3$)	D_t (X^-)	Error	m(X^-)	r(X^-)	V (X^-)	D_t (1^+)	Error	m(1^+)	r(1^+)	V (1^+)
[Code]	[m^2/s]	[%]	[u]	[m^2/s]	[%]	[u]	[A]	[A^3]	[m^2/s]	[%]	[u]	[A]	[A^3]
Acetone	3.05E-09	2.3	-6.00E-03	1.46E-09	1.0	-3.24E-03	3.9	250	1.02E-09	1.2	-2.05E-03	6.1	946
Methanol	2.11E-09	1.5	-4.54E-03	1.05E-09	1.4	-2.21E-03	4.2	306	6.57E-10	0.8	-1.42E-03	6.5	1166
γ-valerolactone	1.24E-09	0.8	-2.64E-03	7.94E-10	2.3	-1.55E-03	4.0	270	3.52E-10	1.4	-8.55E-04	6.3	1067
Propylene carbonate	1.11E-09	1.1	-2.22E-03	9.47E-10	2.5	-1.49E-03	3.7	217	3.56E-10	1.4	-7.37E-04	6.3	1023
p-cymene	1.82E-09	1.6	-3.82E-03	5.26E-10	26.4	-1.07E-03	7.1	1499	3.60E-10	2.5	-8.89E-04	8.4	2509
p-cymene (**1**Cl)	1.78E-09	1.8	-3.73E-03						6.01E-10	0.8	-1.20E-03	6.4	1073

7 References:

(1) Biasiolo, L.; Trinchillo, M.; Belanzoni, P.; Belpassi, L.; Busico, V.; Ciancaleoni, G.; D'Amora, A.; Macchioni, A.; Tarantelli, F.; Zuccaccia, D. Unexpected Anion Effect in the Alkoxylation of Alkynes Catalyzed by N-Heterocyclic Carbene (NHC) Cationic Gold Complexes. *Chem. - Eur. J.* **2014**, *20* (45), 14594–14598. https://doi.org/10.1002/chem.201404539.

(2) Li, D.; Keresztes, I.; Hopson, R.; Williard, P. G. Characterization of Reactive Intermediates by Multinuclear Diffusion-Ordered NMR Spectroscopy (DOSY). *Acc. Chem. Res.* **2009**, *42* (2), 270–280. https://doi.org/10.1021/ar800127e.

(3) Johnson, C. S. Diffusion Ordered Nuclear Magnetic Resonance Spectroscopy: Principles and Applications. *Prog. Nucl. Magn. Reson. Spectrosc.* **1999**, *34* (3–4), 203–256. https://doi.org/10.1016/S0079-6565(99)00003-5.

(4) Stejskal, E. O.; Tanner, J. E. Spin Diffusion Measurements: Spin Echoes in the Presence of a Time-Dependent Field Gradient. *J. Chem. Phys.* **1965**, *42* (1), 288–292. https://doi.org/10.1063/1.1695690.

(5) Sinnaeve, D. The Stejskal–Tanner Equation Generalized for Any Gradient Shape—an Overview of Most Pulse Sequences Measuring Free Diffusion. *Concepts Magn. Reson. Part A* **2012**, *40A* (2), 39–65. https://doi.org/10.1002/cmr.a.21223.

(6) Jerschow, A.; Müller, N. Suppression of Convection Artifacts in Stimulated-Echo Diffusion Experiments. Double-Stimulated-Echo Experiments. *J. Magn. Reson.* **1997**, *125* (2), 372–375. https://doi.org/10.1006/jmre.1997.1123.

(7) Jerschow, A.; Müller, N. Convection Compensation in Gradient Enhanced Nuclear Magnetic Resonance Spectroscopy. *J. Magn. Reson.* **1998**, *132* (1), 13–18. https://doi.org/10.1006/jmre.1998.1400.

(8) Tyrrell, H. J. V.; Harris, K. R. *Diffusion in Liquids: A Theoretical and Experimental Study*; Butterworths monographs in chemistry; Butterworths: London Boston, 1984.

(9) Mills, R. Self-Diffusion in Normal and Heavy Water in the Range 1-45°C. *J. Phys. Chem.* **1973**, *77* (5), 685–688.

(10) Macchioni, A.; Ciancaleoni, G.; Zuccaccia, C.; Zuccaccia, D. Determining Accurate Molecular Sizes in Solution through NMR Diffusion Spectroscopy. *Chem Soc Rev* **2008**, *37* (3), 479–489. https://doi.org/10.1039/B615067P.

(11) Zuccaccia, D.; Belpassi, L.; Tarantelli, F.; Macchioni, A. Ion Pairing in Cationic Olefin−Gold(I) Complexes. *J. Am. Chem. Soc.* **2009**, *131* (9), 3170–3171. https://doi.org/10.1021/ja809998y.

(12) Chen, H. C.; Chen, S. H. Diffusion of Crown Ethers in Alcohols. *J. Phys. Chem.* **1984**, *88* (21), 5118–5121. https://doi.org/10.1021/j150665a063.

(13) Zuccaccia, D.; Macchioni, A. An Accurate Methodology to Identify the Level of Aggregation in Solution by PGSE NMR Measurements: The Case of Half-Sandwich Diamino Ruthenium(II) Salts. *Organometallics* **2005**, *24* (14), 3476–3486. https://doi.org/10.1021/om050145k.
